# The pathogenic germline *ETV4* P433L mutation identified in multiple primary lung cancer affect tumor stem-like property by Wnt/β-catenin pathway

**DOI:** 10.1038/s41419-024-07129-z

**Published:** 2024-10-10

**Authors:** Yu Liu, Lingling Fang, Yalong Wang, Tao Fan, Liyu Wang, Chu Xiao, Ziqin Deng, Wenpeng Cai, Bo Zheng, Junfeng Qiu, Chunxiang Li, Jie He

**Affiliations:** 1 Department of Interventional Therapy, National Cancer Center/National Clinical Research Center for Cancer/Cancer Hospital, Chinese Academy of Medical Sciences and Peking Union Medical College, Beijing, China; 2 Department of Anesthesiology, National Cancer Center/National Clinical Research Center for Cancer/Cancer Hospital, Chinese Academy of Medical Sciences and Peking Union Medical College, Beijing, China; 3 Department of Thoracic Surgery, National Cancer Center/National Clinical Research Center for Cancer/Cancer Hospital, Chinese Academy of Medical Sciences and Peking Union Medical College, Beijing, China; 4 Department of pathology, National Cancer Center/National Clinical Research Center for Cancer/Cancer Hospital, Chinese Academy of Medical Sciences and Peking Union Medical College, Beijing, China; 5 China Economics and Management Academy, Central University of Finance and Economics, Beijing, China

**Keywords:** Oncogenesis, Cell invasion, Cancer epigenetics

## Abstract

The occurrence of multiple primary lung cancer (MPLC) has witnessed a significant surge in recent years within the Chinese population. MPLC is distinguished by its potential genetic susceptibility and notable genetic heterogeneity. Investigating the etiology of MPLC holds substantial clinical importance.The whole genome sequencing (WGS) and genome-wide linkage analysis were performed in a family affected by a dominant form of lung abnormalities. Specifically, five family members were diagnosed with MPLC, while nine members had pulmonary nodules and one normal member. To confirm the potential pathogenic germline mutations sites, Sanger sequencing was performed in an additional 162 MPLC family patients. Furthermore, molecular biology experiments were conducted to investigate the function and the mechanism of the identified pathogenic mutation site in lung cancer A549 and H322, both in vitro and in vivo. Linkage analysis revealed the presence of shared genomic regions among affected family members. Subsequent exome sequencing identified a deleterious variant within these linkage intervals, specifically a heterozygous mutation in ETS-oncogene transcription factors 4 (*ETV4*). This particular variant was found in affected family members at a rate of 13 out of 15 individuals. Furthermore, *ETV4* P433L mutation could be detected in an additional MPLC family patients and mutation frequency was 3.7% (6 out of 162). The *ETV4* P433L mutations site was introduced into lung cancer cell lines, resulting in altered migration and stem-like properties of the cancer cells. Further investigation revealed that the activation of the Wnt/β-catenin signaling pathway, which is associated with stemness, could be attributed to the presence of the *ETV4* P433L mutation, suggesting its involvement in tumor promotion. A novel pathogenic germline mutation, *ETV4* P433L, was identified in a dominant MPLC family, with a mutation rate of 3.7% among MPLC family patients. The *ETV4* P433L mutation was found to impact the stem-like properties and migration of tumors through Wnt/β-catenin signaling pathway.

## Introduction

Multiple primary lung cancer (MPLC) is defined as the simultaneous or sequential development of two or more primary lung cancers in the same patient [1]. Approximately 15% of lung cancer patients exhibit the development of two or more lesions [2], with MPLC being a frequently observed manifestation settings [3–5]. The prevalence of MPLC has exhibited a notable rise and is observed in approximately 0.2–8% of newly diagnosed lung carcinomas because of the utilization of low-dose computed tomography (LDCT) cases [6, 7]. MPLC, or multiple ground glass/lepidic (GG/L) nodules, possesses distinctive genetic characteristics and warrants consideration as an independent disease different from lung cancer [8, 9]. The precise etiology of this disease remain unclear, although prior research has indicated a strong correlation between smoking [10–12], field cancerization [13–15], and familial heredity [16] in the onset of MPLC. Our research team revealed a remarkably high incidence rate of 35.8% (220 out of 625) among first-degree relatives [12]. The 162 cases MPLC were identified among these 220 family history patients [12]. Other studies also support the occurrence of MPLC with family history is considerably greater compared to those without such a history [16, 17]. Hence, the genetic characteristics underlying the majority of familial or sporadic MPLC cases remains elusive and necessitates further investigation.

MPLC exhibits genetic heterogeneity, a significant molecular genetic characteristic. The heterogeneity of MPLC well-known drive mutations in focal areas could reach 80%-100% [18, 19]. The high-throughput sequencing results also revealed distinct clonal characteristics of MPLC among multiple lesions within the same patient [9, 20]. The existence of heterogeneity makes the etiology of MPLC more difficult to determine, thus affecting its diagnosis and treatment. Recent comprehensive studies across various cancer types have revealed that approximately 3.5–8.5% of lung cancer cases possess potentially pathogenic germline mutations [21, 22]. Several genetic variants, such as BRCA2 and CHEK2, as well as germline mutations in the EGFR, specifically the mutations in exon 20 and exon 21, have been strongly associated with an increased risk of lung cancer [23–29]. Previous study demonstrated the simultaneous presence of EGFR, KRAS, TP53, or PARP1 mutations in certain cases of multiple primary lung cancers (MPLCs). The occurrence of concomitant EGFR or KRAS mutations in MPLCs was found to be significantly higher [30–32].The *EGFR*-T790M germline mutation was initially discovered in a European MPLC family and subsequently in several other MPLC cases [33, 34]. Our research group has previously demonstrated that the presence of the *EGFR*-R776H germline mutation and unique EGFR mutation signatures in a cohort of 162 family MPLC patients [27]. Therefore, comprehending the genetic predisposition or pathogenic mutation site is of utmost importance in the screening, prevention, and treatment of patients exhibiting germline pathogenic alterations.

In our study, we examined a dominant heredity MPLC family to investigate the potential pathogenicity of germline mutations. Our analysis revealed the presence of a potential pathogenicity mutation, *ETV4* P433L, in both MPLC (5/5) and pulmonary nodule-affected family members (8/9). ETV4, also referred to PEA3 and E1AF, belongs to the polyomavirus enhancer activator 3 (PEA3) subfamily of ETS-oncogene transcription factors. These transcription factors play a crucial role in both normal physiology processes and pathological conditions [35]. ETV4 is implicated in various stages of organ development and serves as a significant mediator in the formation of tumors and metastasis [36, 37]. Numerous studies have reported the overexpression and pathological involvement of ETV4 in various tumor types, such as prostate, breast, lung, and colon [38]. However, there is currently no available research or report on the impact of the P433L mutation site in *ETV4* for etiology. The purpose of our study was to investigate a newly identified pathogenic germline mutation within MPLC family pedigree and assessed the frequency of the *ETV4* P433L mutation in a cohort of 162 family MPLC patient. Additionally, we examined the biological implications of the *ETV4* P433L mutation in lung cancer through in vitro and in vivo experimentation.

## Materials and methods

### Study design and patient selection

The familial lung cancer pedigree consisted of five individuals who were diagnosed with MPLC, nine individuals with multiple lung nodules and one normal individual. In order to confirm the presence of potential pathogenic germline mutations, the study utilized 162 family probands along with their corresponding noncancerous adjacent tissues (NATs). The criteria for diagnosing and including MPLC cases were previously described in our previous study [39]. The formalin-fixed, paraffin-embedded (FFPE) normal tissues collected after surgical resection were used to identify potential pathogenic germline mutations. Ethical approval was obtained from the medical ethic committee of the National Cancer Center/Cancer Hospital, Chinese Academy of Medical Sciences and Peking Union Medical College. And all the patients provided written informed consent.

### Whole-Genome Sequencing and Transcriptome sequencing

Whole-genome sequencing (WGS) was conducted on peripheral blood DNA samples using CapitalBio Technology (China). The SNV data underwent rigorous filtering, including exclusion of variants present in less than 1% of the 1000 East Asia database, the ESP database, and novel mutations not found in the 1000 database, 1000 genome, and ESP6500 whole exon database. Subsequently, the mutation sites were compared between MPLC patients and individuals with multiple pulmonary nodules, after excluding normal individuals. Common mutations sites were filtered out, and the pathogenetic mutation were predicted using SIFT and Polyphen 2 method. The identification of variants was conducted using GATK-HaplotypeCaller.

The Manta tool was employed for the analysis of genomic data, enabling the identification of structural variation (SV) events such as amplification, deletion, inversion, and translocation. The IAnnotateSV tool was utilized to annotate these variations, with a focus on gene-level annotation. We screen out the SV events by applying screening criteria of rec = 5 in the lung cancer group and rec = 9 in the nodule group, while excluding the control group.

Lentiviral transfection was utilized to construct the vector, wild type ETV4, and mutant type *ETV4*(P433L) lung cell lines, which were then selected for Transcriptome sequencing. Illumina PE150 was used for transcriptome sequencing which was carried out by Novagene (China).

### FFPE tissue DNA extraction and Sanger sequencing

Genomic DNA (gDNA) was extracted from the 162 patients using the GeneRead DNA FFPE Kit (Qiagen, Hilden, Germany) and quantified with Qubit (Life Technologies, Carlsbad, USA) following the manufacturer’s protocol. The mutations were amplified from the gDNA using standard PCR. The gene-specific sense and antisense primers were *ETV4* P433L, F(ACCCTCCTGCCAGTATGAA) and R(CCGAGCATCTGCCTGTAC), *KLHL10* V203I, F(CTCCCGACATGATGAAGC) and R(ACCACCAGGAAGGATTTT), *ASB16* R416*, F(CCCAGCCCGCATTATCACTG) and R(AGCCTGACATGGGGTTCACT). PCR amplification and agarose gel detection were conducted three times. The amplification sequence was detected using Sanger sequencing, and the resulting data were analyzed and displayed using Chromas software.

### Cell culture and function experiment

The lung cancer cell lines A549 and H322 were cultured in RPMI-DMEM medium (HyClone) with 10% FBS(Gibco). The cell lines were incubated at a temperature of 37°C and a CO2 concentration of 5%. Cell proliferation was assessed using the Cell Counting Kit-8 (CCK-8) assays (KGA317, KeyGEN), while migration assays were performed using Transwell chambers (#3422, Corning).

Wound healing assays were conducted utilizing a 6 well plate. In brief, cells were seeded until achieving cell attachment and the formation of a confluent cell layer. Subsequently, the inserts were removed and cells were overplayed with culture medium containing 2% FBS. The cells were then incubated at 37°C for a duration of 24 hours. Images were captured at specified intervals, and the relative area of wound closure was assessed using image J software.

For the tumor sphere formation assay, individual cells were cultured at a density of 1 × 10^3^ cells/ml in ultralow attachment 6-well plates containing serum-free DMEM/F12 (Gibco, Carlsbad, CA, USA) supplemented with 2% (v/v) B27 (Invitrogen, Carlsbad, CA, USA), 20 ng/ml EGF (Sigma, St Louis, MO, USA), 20 ng/ml basic fibroblast growth factor (bFGF, BD Biosciences, CA, USA), and 4 μg/ml Heparin (Sigma, St Louis, MO, USA) for a period of 10 to 14 days. The resulting mammospheres were quantified and imaged using an inverted fluorescence microscope.

### Tumor metastasis and limiting dilution tumor sphere formation assay

In the tumor metastasis experiment, NOD-SCID mice were chosen as the experimental model to investigate tumor metastasis. This investigation occurred 8-12 weeks after the injection of ETV4-wild and mutant P433L cells into the tail vein. The NOD/SCID mice were randomly allocated to various groups (Wild+DMSO, Mutant+DMSO, Wild+XAV-939 and Mutant+ XAV-939) following the subcutaneous transplantation of A549 lung cancer cells into the contralateral flanks of the mice at differing dilutions. Subsequently, the mice were intraperitoneally injected with DMSO and/or XAV-939(15 mg/kg) on a daily basis, as per the protocol of a previous study [40]. The mice were euthanized via cervical dislocation, and the xenograft tumors were subsequently excised, photographed, and tumor volume was calculated using the formula: (length × width^2^)/2. The stem cell frequency was subsequently determined using the online tool provided by https://bioinf.wehi.edu.au/software/elda/ [41].

### Plasmid transfection

The shRNAs were cloned into a GV654 vector (Genechem, Shanghai, China) to induce knockdown of ETV4 expression. Full-length ETV4 wild and mutant cDNA were inserted into a GV492 vector system (GeneChem, China) for the purpose of overexpressing ETV4. The packaging plasmids were transfected into HEK-293T cells using Lipofectamine 3000 (L3000075, Thermo) to generate infectious lentivirus particles. The corresponding empty vectors were employed as controls. Stable cell lines were selected using Puromycin (P9620, Sigma-Aldrich) and G418 (10131035, Thermo) after a period of 1-2 weeks.

### Western blotting and RT-qPCR

Total proteins were quantified for concentration using a BCA protein assay kit (#23227, Thermo). The experimental method employed in a previous study was followed for detailed procdures [42]. Antibodies against ETV4 (Abcam, ab189826) and c-Myc (Abcam, ab32072) were purchased. Additionally, ABCG2 (Cell Signaling Technology, #42078), KLF4 (CST, #12173), β-catenin (CST, #8480) and β-actin (CST, #4970) antibodies were also obtained.

Total RNA was isolated using the TRIzol protocol (Thermo), and cDNA synthesis was performed using the EasyScript All-in-one First step cDNA Synthesis SuperMix for qPCR (TransGen, AE341). The qPCR experiment was conducted utilizing PerfectStart GreenqPCR SuperMix (AQ602, TransGen) and an ABI 7900HT real-time PCR thermocycler (Life Technologies). The obtained gene expression data were normalized to the outcomes of the endogenous control β-actin. The 2^−ΔΔCt^ method was employed, and each sample was analyzed in triplicate. The primer sequences can be found in Supplementary Table 1.

### Bioinformatics analysis

Tumor tissue data of lung cancer patients from The Cancer Genome Atlas (TCGA) were analyzed by GEPIA database [43]. The RNA-seq data were used for the ETV4 overexpressing (vector, wild and mutant) group in shETV4-1 A549 cells and H322 cells. The differential gene were calculated and the criterion for differential genes as a |logFoldchange | >1. The function enrichment results were analyzed by DAVID [44]. The Gene set enrichment analysis (GSEA) was performed using GSEA software and the mRNA expression profile was analyzed. The GSEA, employing the Molecular Signatures Database (MSigDB) C2 KEGG gene sets and Hallmark gene sets [45]. The GSEA functional analysis was applied to all genes in the ETV4 mutation and wild-type cell line transcriptome sequencing. The criteria for significant enrichment were set at an enrichment score (ES) greater than 0.4 and a *P* value < 0.05, following 1000 permutations.

### Statistical analysis

Statistical analyses were carried out using SPSS and GraphPad Prism 7.0 software. Statistical analyses involved the utilization of the Student’s t-test, log-rank test, or Mann-Whitney U test for comparing two groups, while the one-way ANOVA was employed to analyze experimental data comprising three or more groups. The presentation of numerical data was in the form of means ± standard deviation (SD). Statistical significance was determined at the thresholds of *p < 0.05, **p < 0.01, and ***p < 0.001, with the designation of NS indicating non-significant differences.

## Result

### Familial MPLC family pedigree and samples

**Fig. 1:**
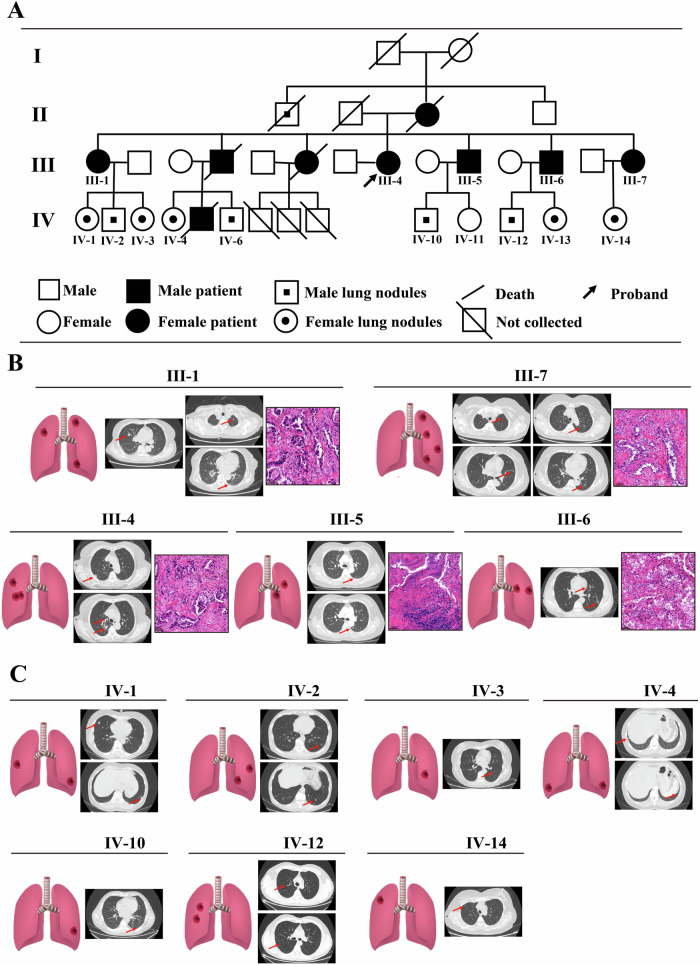
The familial MPLC family pedigree. **A** The familial pedigree of multiple primary lung cancer (MPLC) included five patients (III-1, III-4, III-5, III-6 and III-7) who were diagnosed with MPLC, nine individuals (IV-1, IV-2, IV-3, IV-4, IV-6, IV-10, IV-11, IV-12 and IV-14) with multiple lung nodules, and one normal individual (IV-10). **B** The chest computed tomography (CT) and histopathological examination (HE) results of five MPLC patients were presented. **C** The CT results of patients with lung nodules were presented.

We studied a family with lung nodules included nine patients diagnosed with MPLC and obtained five patient (III-1, III-4, III-5, III-6 and III-7), nine individuals with multiple lung nodules (IV-1, IV-2, IV-3, IV-4, IV-6, IV-10, IV-12, IV-13 and IV-14), and one normal individual blood samples (IV-11) (Fig. 1A). The chest computed tomography (CT) results of five MPLC patients were presented in Fig. 1B. Furthermore, the CT results of patients with lung nodules are depicted in Fig. 1C. These findings suggest that MPLC exhibits familial aggregation, and this familial pedigree holds significant research value and clinical significance.

A cohort of 162 MPLC probands, along with their corresponding NAT samples, were subjected to screening for inheritable pathogenic mutations. The samples were sourced from paraffin-embedded tissues stored in a tumor bank. The clinicopathological characteristics of these probands have been previously described in a study [39].

### Identification and validation of inheritable pathogenic mutations by WGS analysis

Germline mutations were detected through the utilization of whole genome sequencing (WGS) on peripheral blood DNA samples obtained from a cohort of 15 family members. This comprehensive approach allowed for the identification of various types of mutations, including single nucleotide variants (SNV), copy number variants (CNV), structural variants (SV), and insertions/deletions (inDel). The Manta tool was employed for the analysis of genomic data, enabling the identification of structural variation events such as amplification, deletion, inversion, and translocation. The Supplementary Figure 1A illustrates the variation events observed in each sample. Subsequently, the IAnnotateSV tool was utilized to annotate these variations, with a focus on gene-level annotation. By applying screening criteria of rec = 5 in the lung cancer group and rec = 9 in the nodule group, while excluding the control group, a total of 2 mutation events (ARHGAP19 and NRG1) were identified (Supplementary Figure 1B). However, it is important to note that these variants are situated in non-exonic regions.

**Fig. 2:**
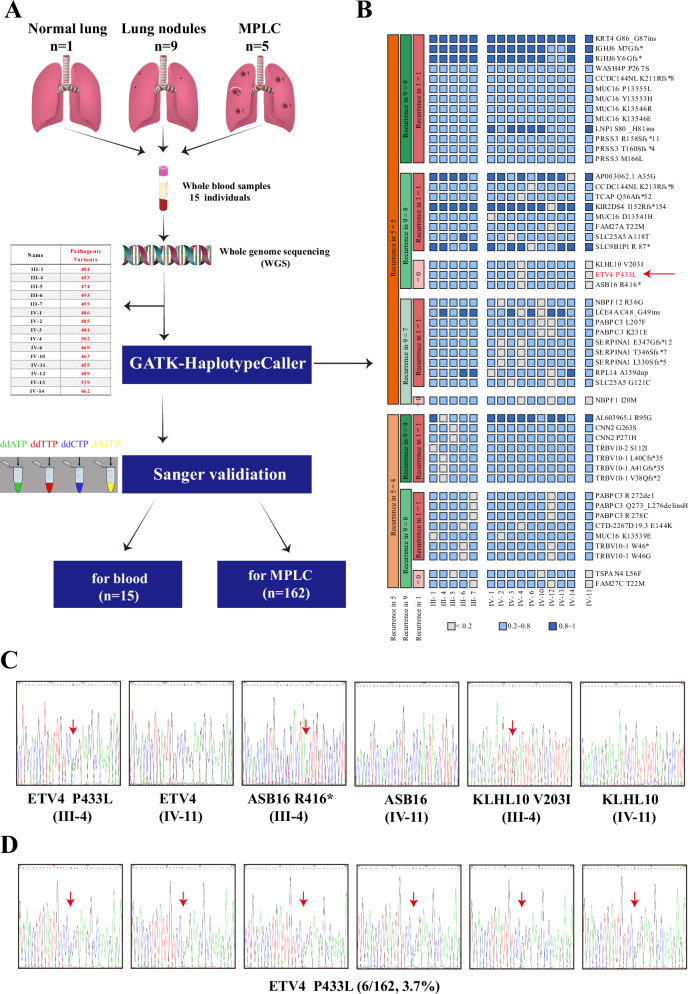
The present of WGS analysis results and verification by Sanger sequencing. **A** The research workflow pertaining to familial MPLC pedigree was conducted. **B** The GATK-HaplotypeCaller method was employed to filter potential pathogenic mutations sites. **C** The verification of *ETV4* P433L, *KLHL10* V203I and *ASB16* R416* in blood DNA of III-4 and IV-11 individuals from the family by Sanger sequencing; **D** Sanger sequencing was employed to verify the presence of *ETV4* P433L in 162 MPLC probands with NATs.

Specifically, for the SNV data, the GATK-HaplotypeCaller method was employed to effectively filter and pinpoint pathogenic mutations. The research workflow pertaining to the investigation of familial MPLC pedigree was visually presented in Fig. 2A. Subsequently, a comparative analysis of mutation sites was conducted between MPLC patients and individuals with multiple lung nodules, after excluding normal individuals from the analysis. We also analyzed the enrichment of common driver gene pathways and results was showed in Supplementary Figure 2. The GATK-HaplotypeCaller method was employed to eliminate numerous prevalent mutation sites, resulting in the identification of three mutation sites (*ETV4* P433L, *KLHL10* V203I, and *ASB16* R416*) present in five MPLC patients and eight lung nodule patients, while no mutations were observed in normal individuals (Fig. 2B).

In order to ascertain the veracity of these mutations, the Sanger sequencing method was utilized to validate them in the blood DNA of 15 family members. Our findings confirmed the concordance between blood DNA and whole-genome sequencing results (Fig. 2C). Furthermore, a cohort of 162 MPLC probands was examined to identify the presence of a specific mutation. Our analysis revealed that the *ETV4* P433L mutation had a rate of 6/162 (3.7%) and demonstrated significant clinical relevance (Fig. 2D).

### The *ETV4* P433L mutation promoted lung cancer cell migration

**Fig. 3:**
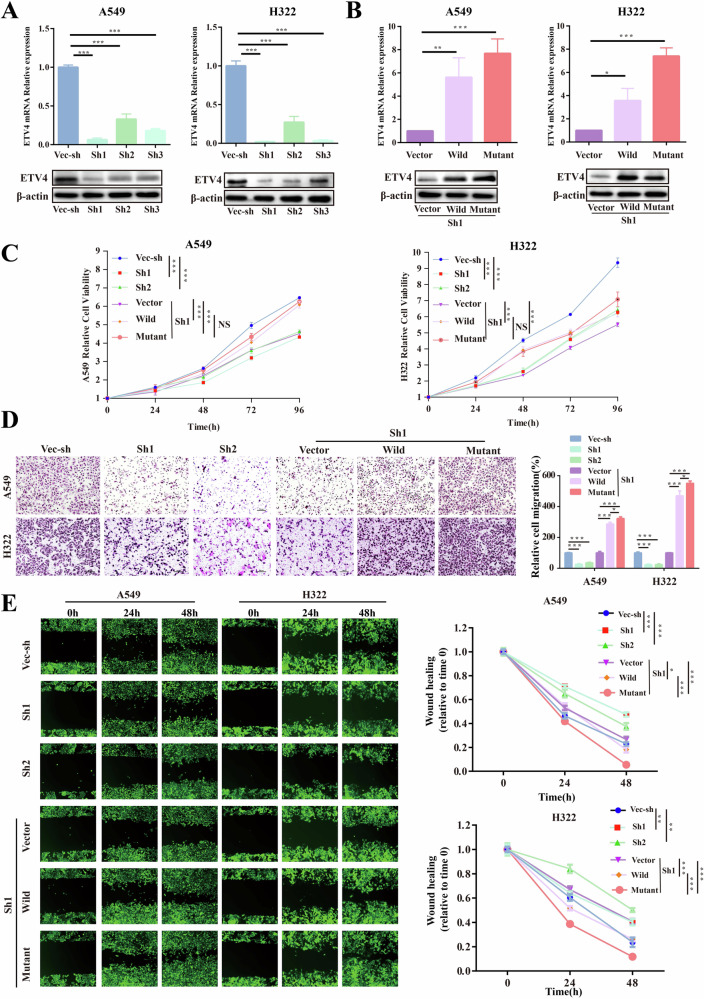
The *ETV4* play a tumor promoting role in lung cancer cell lines. **A**, **B** The construction and identification of ETV4 knockdown and overexpression (wild and mutant) cell lines were performed. **C** The CCK8 cell proliferation assay was conducted for ETV4 knockdown (vec-sh, sh1 and sh2) and overexpression (vector, wild and mutant) cell lines. **D** The Transwell cell migration assay was performed for ETV4 knockdown (vec-sh, sh1 and sh2) and overexpression (Vector, wild and mutant) cell lines. **E** The Wound healing assay was carried out for ETV4 knockdown (vec-sh, sh1 and sh2) and overexpression (vector, wild and mutant) cell lines. Three independent biological replicates were conducted for in vitro assays. The data are presented as the mean ± SD. Statistical significance was determined as *p < 0.05, **p < 0.01 and ***p < 0.001.

The TCGA datasets provided evidence of high expression of ETV4 in lung cancer tissues (Supplementary Fig. 3A). To further investigate the role of ETV4, we conducted knockdown experiments in A549 and H322 cell lines, resulting in reduced expression levels (Fig. 3A and Supplementary Fig. 3B). Notably, our findings demonstrated a significant inhibitory effect on cell proliferation and migration upon ETV4 knockdown (Fig. 3C–E). In order to further investigate the role of the *ETV4* P433L germline mutation in lung cell lines A549 and H322, we constructed wild-type and mutant-type (P433L) ETV4 based on sh1-ETV4 expression downregulated lung cancer cell lines (Fig. 3B and Supplementary Fig. 3C). Our findings demonstrate that the cell proliferation capacity was restored upon upregulation of ETV4 expression, with no significant difference observed between the wild-type and mutant-type ETV4 cells (Fig. 3C). Interestingly, we observed an enhanced cell migration ability in the *ETV4* P433L mutant cell lines compared to the wild-type cell lines (Fig. 3D, E).

### *ETV4* P433L mutation enhance stem-like property of lung cancer cell

**Fig. 4:**
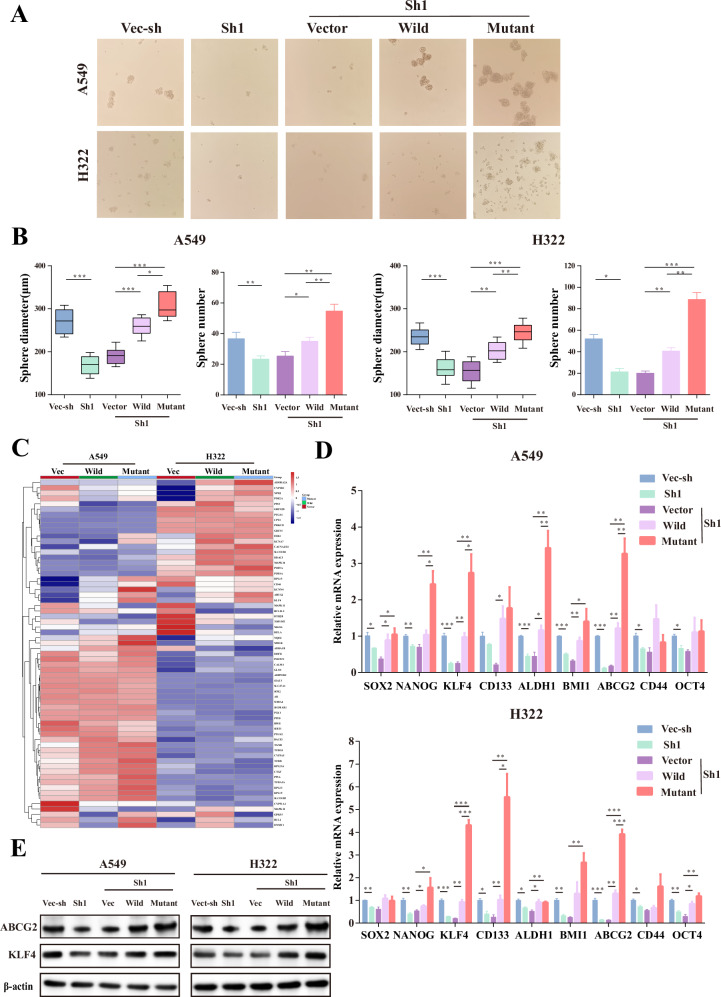
*ETV4* P433L mutation promote stem-like property of lung cancer cell. **A**, **B** The diameter and number of spheres were determined from vec-sh and sh1 A549 cells and H322 cells, as well as vector, wild and mutant ETV4 overexpressing (vector, wild, mutant) in shETV4-1 A549 cells and H322 cells. **C** The heatmap illustrate the intersection of differential genes from A549 and H322 mutated and wild cell lines with stem-related genes through RNA-seq analysis. **D** The RT-qPCR assay revealed the expression of several stem-related genes markers (SOX2, NANOG, KLF4, CD133, ALDH1, BMI1, ABCG2, CD44, OCT4) in knockdown (vec-sh and sh1) and overexpressing (vector, wild and mutant) in shETV4-1 A549 cells and H322 cells. **E** The expression of stem-related gene markers ABCG2 and KLF4 was observed in knockdown (vec-sh and sh1) and overexpressing (vector, wild and mutant) in shETV4-1 A549 cells and H322 cells using Western blot assays. Three independent biological replicates were conducted for in vitro assays. Data are presented as mean ± SD. *p < 0.05, **p < 0.01, ***p < 0.001.

The stem-like property of cancer cells have been linked to the process of tumor metastasis [46, 47]. In order to investigate the potential association between ETV4 expression and the stem cell-like properties of tumors, we performed tumor sphere formation experiments. Our findings revealed that the suppression of ETV4 expression resulted in a decrease in the ability of sphere formation, which could be restored by the overexpression of wild-type ETV4. Notably, the cell lines carrying the *ETV4* P433L mutation exhibited a greater sphere formation ability compared to the cell lines with wild-type ETV4 (Fig. 4A, B). Meanwhile, we aimed to investigate the potential mechanism of wild-type and *ETV4* P433L mutation lung cancer cell lines. We conducted an analysis of differential genes identified through transcriptome sequencing of vector, wild and mutated cell lines in shETV4-1 A549 cells and H322 cells (Supplementary Figure 4A). Our findings revealed a lower number of differential genes within the same cell type (Supplementary Fig. 4B). Consequently, we established the criterion for differential genes as a |log2 Foldchange | >1. Subsequently, an intersection analysis of differential genes present in the two cell lines was conducted to facilitate functional enrichment analysis by DAVID (Supplementary Fig. 4C–E). Consequently, through a comprehensive literature review of tumor stemness genes and tumor stemness index genes (Supplementary Material) [48–51], we cross-referenced these with the differential genes of the two cell lines. The resulting collection is depicted in Fig. 4C and Supplementary Fig. 4F.

To further investigate whether the genes associated with tumor stemness were altered, we selected a subset of representative genes (SOX2, NANOG, KLF4, CD133, ALDH1, BMI1, ABCG2, CD44, and OCT4) for validation in knockdown and overexpression groups using RT-qPCR analysis. Our results demonstrate that the expression levels of various stemness-related genes were reduced in ETV4 knockdown cell lines and elevated in ETV4 overexpression cell lines (Fig. 4D). Notably, among these stemness genes, only ABCG2 and KLF4 were significantly reduced in sh1 compared to vec-sh. Conversely, the overexpression of wild was markedly elevated relative to vector, and the expression of mutant was significantly higher than that of wild. All observed trends were statistically significant (Fig. 4D). Subsequent western blot analysis further validated that the protein levels of ABCG2 and KLF4 were significantly elevated in the mutant cell lines (Fig. 4E and Supplementary Fig. 5A, B). Previous studies have associated ABCG2 and KLF4 with tumor stemness [52–56], suggesting that these genes may serve as representative markers and potentially reflect the disparity in tumor stemness between cell types. The aforementioned findings indicate that the *ETV4* P433L mutation may enhance the stem-like property of lung cancer cells.

### *ETV4* P433L mutation affect stem-like property by Wnt/β-catenin signaling pathway

**Fig. 5:**
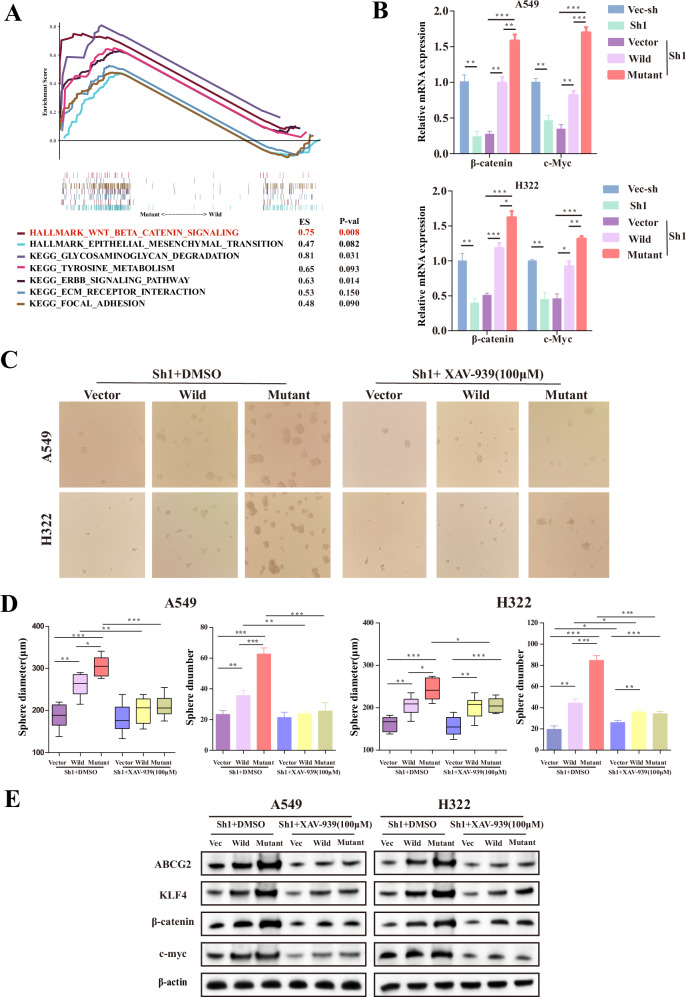
*ETV4* P433L mutation promote stem cell-like property and migration by Wnt/β-catenin signaling pathway. **A** The GSEA function enrichment analysis revealing the enrichment of various tumor-related pathways such as the Wnt/β-catenin signaling pathway, EMT, metabolic pathway, ECM receptor, and focal adhesion. **B** The expression level of β-catenin and c-Myc were assessed in knockdown (vec-sh and sh1) and overexpressing (vector, wild and mutant) in shETV4-1 A549 cells and H322 cell**s** by RT-qPCR. **C**, **D** The diameter and number of spheres derived from shETV4-1 A549 cells and H322 cells overexpression vector, wild-type and *ETV4* P433L mutation (vector, wild and mutant) were measured in the presence or absence of 100 μM XAV-939, an inhibitor of β-catenin signaling. **E** The expression of stem-related gene markers ABCG2, KLF4 and β-catenin and c-Myc in vector, wild-type and *ETV4* P433L mutation cells (vector, wild and mutant) were measured in the presence or absence of 100 μM XAV-939. Three independent biological replicates were conducted for in vitro assays. Data are presented as mean ± SD. *p < 0.05, **p < 0.01, ***p < 0.001.

In order to investigate the impact of the *ETV4* P433L mutation on cell migration and stem-like properties, the GSEA function enrichment analysis was employed, revealing the enrichment of various tumor-related pathways such as the Wnt/β-catenin signaling pathway, EMT, metabolic pathway, ECM receptor, and focal adhesion (Fig. 5A). The association between the Wnt/β-catenin signaling pathway and tumor stem-like properties prompted us to investigate the expression levels of β-catenin and its target gene c-Myc in various cell lines, including vector, wild-type, and *ETV4* P433L mutation cell lines. Our analysis revealed that the expression of both β-catenin and c-Myc was significantly higher in wild-type ETV4 cells compared to vector cells, and in *ETV4* P433L mutant cells compared to wild-type ETV4 cells (Fig. 5B). To further elucidate the involvement of the Wnt/β-catenin pathway in this process, we employed the selective inhibitor XAV-939 (100 μM) to inhibit Wnt/β-catenin mediated transcription. Our findings indicate that this inhibition led to a reduction in the tumor sphere formation ability of *ETV4* P433L mutation cells (Fig. 5C, D). Additionally, the expression levels of stemness markers ABCG2, KLF4, and the target gene c-Myc were also diminished in *ETV4* P433L mutation cells (Fig. 5E and Supplementary Fig. 6A, B). These observations collectively suggest that the *ETV4* P433L mutation may regulate the stem-like properties and migration of lung cancer cells through the Wnt/β-catenin signaling pathway.

### *ETV4* P433L mutation promote tumor sphere formation and tumor metastasis

**Fig. 6:**
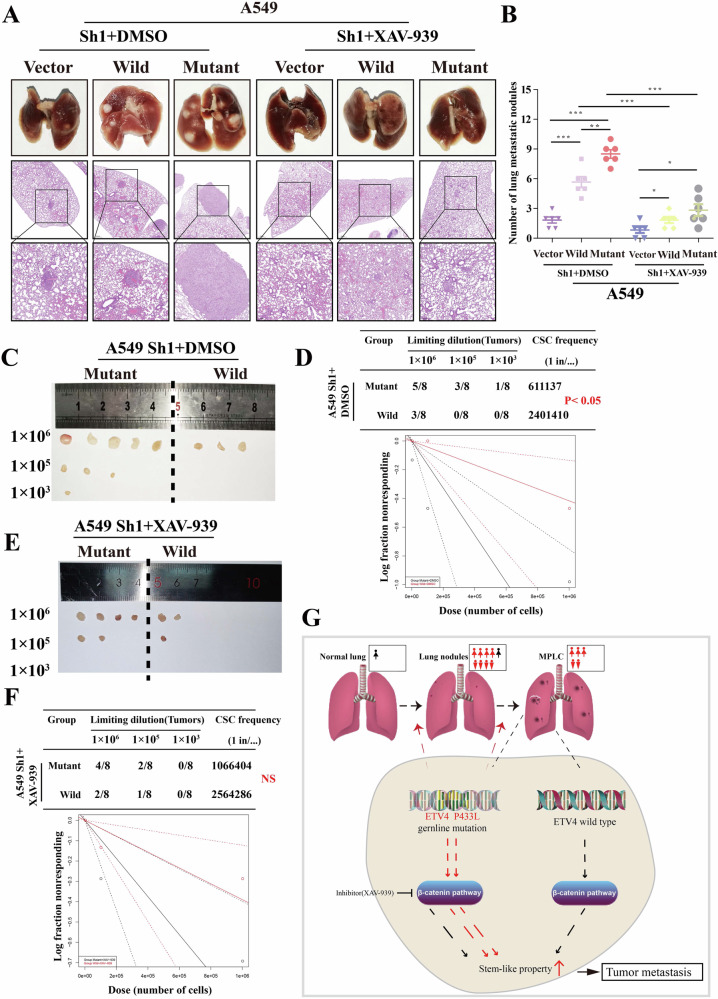
*ETV4* P433L mutation promote stem cell-like property and metastasis in vivo and the graphical summary. **A** Representative images of the gross lesion in lung tissues and hematoxylin and eosin staining of metastatic nodules in the lungs from the vector, wild-type and *ETV4* P433L mutation cells were measured in the presence or absence of XAV-939 (15 mg/kg). **B** The quantification results of metastatic nodule numbers are displayed. **C–F** In vivo limited dilution assays were conducted for the A549 cell lines, revealing representative tumor incidence and CSC probabilities from the wild-type and *ETV4* P433L mutation cells were measured in the presence or absence of XAV-939 (15 mg/kg). **G** The graphical summary of the function and mechanism of *ETV4* P433L mutation. *p < 0.05, **p < 0.01, ***p < 0.001.

We conducted a comparative analysis of the metastatic potential of cancer cells through lung cancer colonization. Our findings revealed that the *ETV4* P433L mutation cells exhibited a greater capacity for lung tumor colonization in comparison to the wild type cell lines. Then, when the inhibitor XAV-939 was added, this effect was significantly reduced (Fig. 6A, B). In order to assess the stem-like property of cancer cells, we employed in vivo limited dilution tumor sphere formation assays. Specifically, we introduced varying concentrations of ETV4 wild type and P433L mutation A549 cell lines into subcutaneous NOD/SCID mice in the presence or absence of XAV-939 (15 mg/kg). As anticipated, the presence of the *ETV4* P433L mutation in cancer cells led to a higher incidence of tumor formation compared to the wild type cell lines, the inhibitor XAV-939 could also significantly inhibit this effect (Fig. 6C–F).

## Discussion

Lung cancer is a prevalent form of cancer worldwide, with genetic factors playing a significant role in its development [57]. Precision therapy for lung cancer often targets EGFR, although limited evidence exists regarding the involvement of EGFR germline mutations in MPLC [25, 26, 28, 29]. For example, the *EGFR*-T790M and *EGFR*-R776H germline mutation were discovered in MPLC cases patients [27, 33, 34]. These results indicated the MPLC has a genetic component, but the cause is unknown. Our previous research has demonstrated the presence of EGFR mutations in MPLC patients [27, 39], and found a dominant heredity familial MPLC pedigree. These results indicated the presence of genetic factors and the family study was a good method to study the etiology of hereditary MPLC. In light of the potential genetic mutation of pathogenicity, our research has identified the *ETV4* P433L mutation site as a potential pathogenicity mutation within the MPLC family population. Furthermore, we have observed this mutation in sporadic cases of MPLC and have determined the mutation frequency of the *ETV4* P433L site to be 3.7% (6/162). These finding indicate a familial aggregation of MPLC and suggest that the key germline mutation site *ETV4* P433L may play a significant role in the development of MPLC, thereby possessing valuable clinical implications.

Multiple studies have indicated a low prevalence of EGFR germline mutation in lung cancer, yet it has been directly associated with tumorigenesis [25–29, 39]. For instance, a comprehensive study revealed the presence of *EGFR* p.T790M germline mutation (0.15%) in plasma Cell-free DNA (cfDNA) from lung cancer [58]. However, our investigation of this MPLC family did not uncover any potential pathogenic EGFR mutations. Intriguingly, WGS analysis identified potential pathogenic mutations in *KLHL10*, *ETV4* and *ASB16*, located at positions 40 M, 41 M and 42 M on chromosome 17, respectively, suggesting potential linked mutation events. Therefore, our study aimed to investigate the presence of *KLHL10*, *ETV4* and *ASB16* mutant sites in a cohort of 162 sporadic MPLC patients. However, our findings only revealed the presence of the *ETV4* P433L mutation, with a mutation frequency of 3.7% (6/162). While this result does not dismiss the potential involvement of KLHL10 and ASB16 mutations in this patient population, it does suggest a strong association between the *ETV4* P433L mutation and MPLC. Previous pan-cancer studies have reported a prevalence of 3.5-8.5% of lung cancers harboring likely pathogenic germline mutations(21, 22]. A study conducted on 1794 Chinese lung cancer patients revealed a pathogenic or likely pathogenic(P/LP) germline mutation rate of 5.91% (106/1794) [59]. In our own study, we observed a mutation frequency of 3.7% (6/162) at the *ETV4* site in patients with MPLC, which aligns with the findings of previous studies. Notably, this frequency is significantly higher than the currently known mutation rate of MPLC *EGFR* p.T790M(0.15%), suggesting that further investigation into the ETV4 site holds greater potential for exploration.

ETS variant 4 (ETV4), a member of the polyoma enhancer activator protein (PEA3) subgroup [35, 60], has been found to exhibit abnormal expression in various tumors, thereby facilitating tumor development and metastasis [61–64]. Previous studies have demonstrated that the loss of ETV4 significantly hampers the expression of glycolytic enzymes, diminishes glucose uptake, and reduces lactate release in breast cancer cells [65]. Furthermore, ETV4 plays a crucial role in activating the Sonic Hedgehog signaling, which is associated with the maintenance of breast cancer stemness [65]. The similar result was also showed that ETV4 facilitated the development of malignant tumor characteristics by stabilizing β-catenin expression in the nucleus and synergistically interacting with the PI3K/Akt pathway to activate Wnt/β-catenin [66]. Our investigation confirmed that suppressing ETV4 expression in lung cancer cell lines significantly decreased tumor proliferation and migration ability, and downregulated the expression of stemness genes. Furthermore, we investigated the impact of the *ETV4* P433L mutation and observed an increase in migration and cell stem-like properties, but not tumor proliferation (Fig. 3C and Supplementary Figure 7A-C).Notch, Hedgehog, and Wnt pathway are highly conserved signaling pathways that has significant implications for tumor homeostasis and stenness [67, 68]. Specifically, the Wnt/β-catenin pathway, which is considered a classic stemness pathway, is closely associated with stem-like alterations in tumors [67, 68]. Our findings further support the notion that activation of Wnt/β-catenin pathway in *ETV4* P433L mutation cells promotes the acquisition of stem-like properties in lung cancer cells compared to wild-type ETV4 cell (Fig. 6G). Prior research has demonstrated that ETV4 can bind to the promoters of its downstream targets, thereby facilitating the transcriptional activation of their expression. For instance, ETV4 has been found to interact with ANXA2, thereby enhancing the activation of the Wnt/β-catenin pathway [69]. The expression of components and regulators of the Wnt/β-catenin pathway, such as TCF4, LPR6, GSK3β, and DKK4, can also be influenced by ETV4 [70, 71]. Furthermore, ETV4 may influence the stabilization of nuclear β-catenin by promoting its interaction with COP1, thereby facilitating β-catenin’s proteasomal degradation [70]. Additionally, ETV4 and β-catenin may enhance the stability of associated regulatory proteins and c-Jun signaling, which has been documented to stabilize β-catenin [72]. Consequently, we hypothesize that the effects of ETV4 on β-catenin may operate through these mechanisms. Nonetheless, from the standpoint of the gene itself, the alterations in the *ETV4* gene following the P433L point mutation remain ambiguous. These changes may potentially influence the transcription and translation of ETV4 itself, and may also entail modifications in the ETV4 protein structure. These alterations could potentially impact the selection of downstream target genes and pathways as transcription factors, and the manner in which it exerts its regulatory function. These aspects will be the subject of further investigation in future studies. These results suggest that ETV4 serves as a crucial upstream transcriptional regulator in lung cancer. However, our study has yet to establish a definitive link between the *ETV4* P433L mutation and the presence of MPLC or multiple lung nodes.

However, this study has several limitations that should be acknowledged. Firstly, it is important to note that the 162 patients included in our study were all diagnosed with multiple primary lung cancers (MPLC). Therefore, it remains uncertain whether the *ETV4* P433L mutation is exclusively associated with MPLC or if it also plays a role in other types of lung cancer. To address this uncertainty, further investigation with a larger verification cohort is necessary to determine the mutation frequency specifically in MPLC patients. Secondly, the examination of the functional implications of the *ETV4* P433L mutation in this study represents only a preliminary exploration. The impact of this mutation on the structure of the ETV4 protein and its ability to activate key sites within downstream pathways remains elusive. Further research is required to elucidate these aspects in order to gain a comprehensive understanding of the mutation’s functional consequences. In our study, we observed the presence of the *ETV4* P433L mutation in 8 patients with lung nodules and 5 patients with MPLC. This finding suggests that the *ETV4* P433L mutation may play a role not only in the development and progression of tumors, but also in the transformation of benign tumors into malignant ones. Further investigations will be conducted in subsequent studies to delve deeper into this phenomenon.

In summary, our study identified a novel pathogenic germline mutation *ETV4* P433L in a dominant MPLC family and displayed a 3.7% mutation rate in MPLC family patients. The *ETV4* P433L mutation could impact the stem-like properties and migration of lung cancer through Wnt/β-catenin signaling pathway.

## Availability of data and material

The WGS data has been uploaded to the National Genome Data Center. The datasets used and/or analyzed during the current study are available from the corresponding author on reasonable request.

## Supplementary information

Supplementary Material

Supplementary Figure

Supplementary Table

## References

[1] Martini N, Melamed MR. Multiple primary lung cancers. J Thorac cardiovascular Surg. 1975;70:606–12. 170482

[2] Murphy SJ, Harris FR, Kosari F, Barreto Siqueira Parrilha Terra S, Nasir A, Johnson SH, et al. Using genomics to differentiate multiple primaries from metastatic lung cancer. J Thorac Oncol: Off Publ Int Assoc Study Lung Cancer. 2019;14:1567–82. 10.1016/j.jtho.2019.05.008 31103780

[3] Leventakos K, Peikert T, Midthun DE, Molina JR, Blackmon S, Nichols FC, et al. Management of Multifocal Lung Cancer: Results of a Survey. J Thorac Oncol: Off Publ Int Assoc Study Lung Cancer. 2017;12:1398–402. 10.1016/j.jtho.2017.05.013 PMC5665365 28583587

[4] Zheng R, Shen Q, Mardekian S, Solomides C, Wang ZX, Evans NR 3rd. Molecular profiling of key driver genes improves staging accuracy in multifocal non-small cell lung cancer. J Thorac cardiovascular Surg. 2020;160:e71–e9. 10.1016/j.jtcvs.2019.11.126 32007245

[5] Jiang L, He J, Shi X, Shen J, Liang W, Yang C, et al. Prognosis of synchronous and metachronous multiple primary lung cancers: systematic review and meta-analysis. Lung cancer (Amst, Neth). 2015;87:303–10. 10.1016/j.lungcan.2014.12.013 25617985

[6] Herbst RS, Morgensztern D, Boshoff C. The biology and management of non-small cell lung cancer. Nature. 2018;553:446–54. 29364287 10.1038/nature25183

[7] Jung EJ, Lee JH, Jeon K, Koh WJ, Suh GY, Chung MP, et al. Treatment outcomes for patients with synchronous multiple primary non-small cell lung cancer. Lung cancer (Amst, Neth). 2011;73:237–42. 10.1016/j.lungcan.2010.11.008 21145616

[8] Lim W, Ridge CA, Nicholson AG, Mirsadraee S. The 8(th) lung cancer TNM classification and clinical staging system: review of the changes and clinical implications. Quant imaging Med Surg. 2018;8:709–18. 30211037 10.21037/qims.2018.08.02 PMC6127520

[9] Park E, Ahn S, Kim H, Park SY, Lim J, Kwon HJ, et al. Targeted Sequencing Analysis of Pulmonary Adenocarcinoma with Multiple Synchronous Ground-Glass/Lepidic Nodules. J Thorac Oncol: Off Publ Int Assoc Study Lung Cancer. 2018;13:1776–83. 10.1016/j.jtho.2018.07.097 30121391

[10] Tucker MA, Murray N, Shaw EG, Ettinger DS, Mabry M, Huber MH, et al. Second primary cancers related to smoking and treatment of small-cell lung cancer. Lung Cancer Working Cadre. J Natl Cancer Inst. 1997;89:1782–8. 9392619 10.1093/jnci/89.23.1782

[11] Strong MS, Incze J, Vaughan CW. Field cancerization in the aerodigestive tract–its etiology, manifestation, and significance. J Otolaryngol. 1984;13:1–6. 6716542

[12] Zhang Z, Gao S, Mao Y, Mu J, Xue Q, Feng X, et al. Surgical outcomes of synchronous multiple primary non-small cell lung cancers. Sci Rep. 2016;6:23252. 27254665 10.1038/srep23252 PMC4890551

[13] Braakhuis BJ, Tabor MP, Kummer JA, Leemans CR, Brakenhoff RH. A genetic explanation of Slaughter’s concept of field cancerization: evidence and clinical implications. Cancer Res. 2003;63:1727–30. 12702551

[14] Johnson BE. Second lung cancers in patients after treatment for an initial lung cancer. J Natl Cancer Inst. 1998;90:1335–45. 9747865 10.1093/jnci/90.18.1335

[15] Li X, Hemminki K. Familial and second lung cancers: a nation-wide epidemiologic study from Sweden. Lung cancer (Amst, Neth). 2003;39:255–63. 10.1016/s0169-5002(02)00535-4 12609563

[16] Li X, Hemminki K. Familial multiple primary lung cancers: a population-based analysis from Sweden. Lung cancer (Amst, Neth). 2005;47:301–7. 10.1016/j.lungcan.2004.07.048 15713513

[17] Haraguchi S, Koizumi K, Hioki M, Hisayoshi T, Hirata T, Shimizu K. Hereditary factors in multiple primary malignancies associated with lung cancer. Surg today. 2007;37:375–8. 17468817 10.1007/s00595-006-3420-5

[18] Chen K, Chen W, Cai J, Yang F, Lou F, Wang X, et al. Favorable prognosis and high discrepancy of genetic features in surgical patients with multiple primary lung cancers. J Thorac cardiovascular Surg. 2018;155:371–9.e1. 10.1016/j.jtcvs.2017.08.141 29092754

[19] Wu C, Zhao C, Yang Y, He Y, Hou L, Li X, et al. High Discrepancy of Driver Mutations in Patients with NSCLC and Synchronous Multiple Lung Ground-Glass Nodules. J Thorac Oncol: Off Publ Int Assoc Study Lung Cancer. 2015;10:778–83. 10.1097/JTO.0000000000000487 25629635

[20] Liu Y, Zhang J, Li L, Yin G, Zhang J, Zheng S, et al. Genomic heterogeneity of multiple synchronous lung cancer. Nat Commun. 2016;7:13200. 27767028 10.1038/ncomms13200 PMC5078731

[21] Huang KL, Mashl RJ, Wu Y, Ritter DI, Wang J, Oh C, et al. Pathogenic Germline Variants in 10,389 Adult Cancers. Cell. 2018;173:355–70.e14. 29625052 10.1016/j.cell.2018.03.039 PMC5949147

[22] Lu C, Xie M, Wendl MC, Wang J, McLellan MD, Leiserson MD, et al. Patterns and functional implications of rare germline variants across 12 cancer types. Nat Commun. 2015;6:10086. 26689913 10.1038/ncomms10086 PMC4703835

[23] Prudkin L, Tang X, Wistuba II. Germ-line and somatic presentations of the EGFR T790M mutation in lung cancer. J Thorac Oncol: Off Publ Int Assoc Study Lung Cancer. 2009;4:139–41. 10.1097/JTO.0b013e3181915f92 PMC4512946 19096324

[24] Tibaldi C, Giovannetti E, Vasile E, Boldrini L, Gallegos-Ruiz MI, Bernardini I, et al. Inherited germline T790M mutation and somatic epidermal growth factor receptor mutations in non-small cell lung cancer patients. J Thorac Oncol: Off Publ Int Assoc Study Lung Cancer. 2011;6:395–6. 10.1097/JTO.0b013e3182059a6f 21252721

[25] Oxnard GR, Miller VA, Robson ME, Azzoli CG, Pao W, Ladanyi M, et al. Screening for germline EGFR T790M mutations through lung cancer genotyping. J Thorac Oncol: Off Publ Int Assoc Study Lung Cancer. 2012;7:1049–52. 10.1097/JTO.0b013e318250ed9d PMC3354706 22588155

[26] van Noesel J, van der Ven WH, van Os TA, Kunst PW, Weegenaar J, Reinten RJ, et al. Activating germline R776H mutation in the epidermal growth factor receptor associated with lung cancer with squamous differentiation. J Clin Oncol: Off J Am Soc Clin Oncol. 2013;31:e161–4. 10.1200/JCO.2012.42.1586 23358982

[27] Su K, Gao S, Ying J, Zou S, He J. Sequencing a super multiple synchronous lung cancer reveals a novel variant in driver gene ARID1B. J Thorac cardiovascular Surg. 2018;155:e185–e91. 10.1016/j.jtcvs.2018.01.010 29576263

[28] Ikeda K, Nomori H, Mori T, Sasaki J, Kobayashi T. Novel germline mutation: EGFR V843I in patient with multiple lung adenocarcinomas and family members with lung cancer. Ann Thorac Surg. 2008;85:1430–2. 18355544 10.1016/j.athoracsur.2007.10.012

[29] Ohtsuka K, Ohnishi H, Kurai D, Matsushima S, Morishita Y, Shinonaga M, et al. Familial lung adenocarcinoma caused by the EGFR V843I germ-line mutation. J Clin Oncol: Off J Am Soc Clin Oncol. 2011;29:e191–2. 10.1200/JCO.2010.31.4492 21172876

[30] Izumi M, Oyanagi J, Sawa K, Fukui M, Ogawa K, Matsumoto Y, et al. Mutational landscape of multiple primary lung cancers and its correlation with non-intrinsic risk factors. Sci Rep. 2021;11:5680. 33707471 10.1038/s41598-021-83609-y PMC7952588

[31] Pei G, Li M, Min X, Liu Q, Li D, Yang Y, et al. Molecular identification and genetic characterization of early-stage multiple primary lung cancer by large-panel next-generation sequencing analysis. Front Oncol. 2021;11:653988. 34109114 10.3389/fonc.2021.653988 PMC8183821

[32] Wang Y, Wang G, Zheng H, Liu J, Ma G, Huang G, et al. Distinct gene mutation profiles among multiple and single primary lung adenocarcinoma. Front Oncol. 2022;12:1014997. 36531058 10.3389/fonc.2022.1014997 PMC9755731

[33] Bell DW, Gore I, Okimoto RA, Godin-Heymann N, Sordella R, Mulloy R, et al. Inherited susceptibility to lung cancer may be associated with the T790M drug resistance mutation in EGFR. Nat Genet. 2005;37:1315–6. 16258541 10.1038/ng1671

[34] Lou Y, Pecot CV, Tran HT, DeVito VJ, Tang XM, Heymach JV, et al. Germline mutation of T790M and dual/multiple EGFR mutations in patients with lung adenocarcinoma. Clin Lung Cancer. 2016;17:e5–11. 26700910 10.1016/j.cllc.2015.11.003 PMC5119523

[35] Oh S, Shin S, Janknecht R. ETV1, 4 and 5: an oncogenic subfamily of ETS transcription factors. Biochimica et biophysica acta. 2012;1826:1–12. 22425584 10.1016/j.bbcan.2012.02.002 PMC3362686

[36] de Launoit Y, Baert JL, Chotteau-Lelievre A, Monte D, Coutte L, Mauen S, et al. The Ets transcription factors of the PEA3 group: transcriptional regulators in metastasis. Biochimica et biophysica acta. 2006;1766:79–87. 16546322 10.1016/j.bbcan.2006.02.002

[37] Laudet V, Hänni C, Stéhelin D, Duterque-Coquillaud M. Molecular phylogeny of the ETS gene family. Oncogene. 1999;18:1351–9. 10022817 10.1038/sj.onc.1202444

[38] Plotnik JP, Budka JA, Ferris MW, Hollenhorst PC. ETS1 is a genome-wide effector of RAS/ERK signaling in epithelial cells. Nucleic acids Res. 2014;42:11928–40. 25294825 10.1093/nar/gku929 PMC4231772

[39] Li C, Wang Y, Su K, Liu Y, Wang L, Zheng B, et al. Presentation of EGFR mutations in 162 family probands with multiple primary lung cancer. Transl lung cancer Res. 2021;10:1734–46. 34012789 10.21037/tlcr-20-1001 PMC8107753

[40] Wang Y, Gao G, Wei X, Zhang Y, Yu J. UBE2T promotes temozolomide resistance of glioblastoma through regulating the Wnt/β-catenin signaling pathway. Drug Des, Dev Ther. 2023;17:1357–69. 10.2147/DDDT.S405450 PMC10168001 37181827

[41] Hu Y, Smyth GK. ELDA: extreme limiting dilution analysis for comparing depleted and enriched populations in stem cell and other assays. J immunological methods. 2009;347:70–8. 19567251 10.1016/j.jim.2009.06.008

[42] Liu Y, Li C, Fang L, Wang L, Liu H, Tian H, et al. Lipid metabolism-related lncRNA SLC25A21-AS1 promotes the progression of oesophageal squamous cell carcinoma by regulating the NPM1/c-Myc axis and SLC25A21 expression. Clin Transl Med. 2022;12:e944. 35735113 10.1002/ctm2.944 PMC9218933

[43] Tang Z, Li C, Kang B, Gao G, Li C, Zhang Z. GEPIA: a web server for cancer and normal gene expression profiling and interactive analyses. Nucleic acids Res. 2017;45:W98–w102. 28407145 10.1093/nar/gkx247 PMC5570223

[44] Sherman BT, Hao M, Qiu J, Jiao X, Baseler MW, Lane HC, et al. DAVID: a web server for functional enrichment analysis and functional annotation of gene lists (2021 update). Nucleic acids Res. 2022;50:W216–w21. 35325185 10.1093/nar/gkac194 PMC9252805

[45] Subramanian A, Tamayo P, Mootha VK, Mukherjee S, Ebert BL, Gillette MA, et al. Gene set enrichment analysis: a knowledge-based approach for interpreting genome-wide expression profiles. Proc Natl Acad Sci USA. 2005;102:15545–50. 16199517 10.1073/pnas.0506580102 PMC1239896

[46] Pattabiraman DR, Weinberg RA. Tackling the cancer stem cells - what challenges do they pose? Nat Rev Drug Discov. 2014;13:497–512. 24981363 10.1038/nrd4253 PMC4234172

[47] Lytle NK, Barber AG, Reya T. Stem cell fate in cancer growth, progression and therapy resistance. Nat Rev Cancer. 2018;18:669–80. 30228301 10.1038/s41568-018-0056-x PMC8388042

[48] Malta TM, Sokolov A, Gentles AJ, Burzykowski T, Poisson L, Weinstein JN, et al. Machine Learning Identifies Stemness Features Associated with Oncogenic Dedifferentiation. Cell. 2018;173:338–54.e15. 29625051 10.1016/j.cell.2018.03.034 PMC5902191

[49] Ding K, Jiang X, Ni J, Zhang C, Li A, Zhou J. JWA inhibits nicotine-induced lung cancer stemness and progression through CHRNA5/AKT-mediated JWA/SP1/CD44 axis. Ecotoxicol Environ Saf. 2023;259:115043. 37224781 10.1016/j.ecoenv.2023.115043

[50] Bu J, Zhang Y, Wu S, Li H, Sun L, Liu Y, et al. KK-LC-1 as a therapeutic target to eliminate ALDH(+) stem cells in triple negative breast cancer. Nat Commun. 2023;14:2602. 37147285 10.1038/s41467-023-38097-1 PMC10163259

[51] Liu B, Fang X, Kwong DL, Zhang Y, Verhoeft K, Gong L, et al. Targeting TROY-mediated P85a/AKT/TBX3 signaling attenuates tumor stemness and elevates treatment response in hepatocellular carcinoma. J Exp Clin cancer Res: CR. 2022;41:182. 35610614 10.1186/s13046-022-02401-6 PMC9131684

[52] Bandey I, Chiou SH, Huang AP, Tsai JC, Tu PH. Progranulin promotes Temozolomide resistance of glioblastoma by orchestrating DNA repair and tumor stemness. Oncogene. 2015;34:1853–64. 24793792 10.1038/onc.2014.92

[53] Zhang W, Mojsilovic-Petrovic J, Andrade MF, Zhang H, Ball M, Stanimirovic DB. The expression and functional characterization of ABCG2 in brain endothelial cells and vessels. FASEB J : Off Publ Federation Am Societies Exp Biol. 2003;17:2085–7. 10.1096/fj.02-1131fje 12958161

[54] Bleau AM, Hambardzumyan D, Ozawa T, Fomchenko EI, Huse JT, Brennan CW, et al. PTEN/PI3K/Akt pathway regulates the side population phenotype and ABCG2 activity in glioma tumor stem-like cells. cell stem cell. 2009;4:226–35. 19265662 10.1016/j.stem.2009.01.007 PMC3688060

[55] Takahashi K, Yamanaka S. Induction of pluripotent stem cells from mouse embryonic and adult fibroblast cultures by defined factors. Cell. 2006;126:663–76. 16904174 10.1016/j.cell.2006.07.024

[56] Yu J, Vodyanik MA, Smuga-Otto K, Antosiewicz-Bourget J, Frane JL, Tian S, et al. Induced pluripotent stem cell lines derived from human somatic cells. Sci (N. Y, NY). 2007;318:1917–20. 10.1126/science.1151526 18029452

[57] Tomoshige K, Matsumoto K, Tsuchiya T, Oikawa M, Miyazaki T, Yamasaki N, et al. Germline mutations causing familial lung cancer. J Hum Genet. 2015;60:597–603. 26178433 10.1038/jhg.2015.75

[58] Hu Y, Alden RS, Odegaard JI, Fairclough SR, Chen R, Heng J, et al. Discrimination of Germline EGFR T790M Mutations in Plasma Cell-Free DNA Allows Study of Prevalence Across 31,414 Cancer Patients. Clin Cancer Res: Off J Am Assoc Cancer Res. 2017;23:7351–9. 10.1158/1078-0432.CCR-17-1745 PMC5712272 28947568

[59] Peng W, Li B, Li J, Chang L, Bai J, Yi Y, et al. Clinical and genomic features of Chinese lung cancer patients with germline mutations. Nat Commun. 2022;13:1268. 35273153 10.1038/s41467-022-28840-5 PMC8913621

[60] Sharrocks AD. The ETS-domain transcription factor family. Nat Rev Mol cell Biol. 2001;2:827–37. 11715049 10.1038/35099076

[61] Wang Y, Ding X, Liu B, Li M, Chang Y, Shen H, et al. ETV4 overexpression promotes progression of non-small cell lung cancer by upregulating PXN and MMP1 transcriptionally. Mol carcinogenesis. 2020;59:73–86. 10.1002/mc.23130 31670855

[62] Kim E, Kim D, Lee JS, Yoe J, Park J, Kim CJ, et al. Capicua suppresses hepatocellular carcinoma progression by controlling the ETV4-MMP1 axis. Hepatol (Baltim, Md). 2018;67:2287–301. 10.1002/hep.29738 29251790

[63] Xu L, Hu H, Zheng LS, Wang MY, Mei Y, Peng LX, et al. ETV4 is a theranostic target in clear cell renal cell carcinoma that promotes metastasis by activating the pro-metastatic gene FOSL1 in a PI3K-AKT dependent manner. Cancer Lett. 2020;482:74–89. 32305558 10.1016/j.canlet.2020.04.002

[64] Gao X, Jiang M, Chu Y, Han Y, Jin Y, Zhang W, et al. ETV4 promotes pancreatic ductal adenocarcinoma metastasis through activation of the CXCL13/CXCR5 signaling axis. Cancer Lett. 2022;524:42–56. 34582976 10.1016/j.canlet.2021.09.026

[65] Zhu T, Zheng J, Zhuo W, Pan P, Li M, Zhang W, et al. ETV4 promotes breast cancer cell stemness by activating glycolysis and CXCR4-mediated sonic Hedgehog signaling. Cell death Discov. 2021;7:126. 34052833 10.1038/s41420-021-00508-x PMC8164634

[66] Zeng S, Seifert AM, Zhang JQ, Kim TS, Bowler TG, Cavnar MJ, et al. ETV4 collaborates with Wnt/β-catenin signaling to alter cell cycle activity and promote tumor aggressiveness in gastrointestinal stromal tumor. Oncotarget. 2017;8:114195–209. 29371979 10.18632/oncotarget.23173 PMC5768396

[67] Takebe N, Miele L, Harris PJ, Jeong W, Bando H, Kahn M, et al. Targeting Notch, Hedgehog, and Wnt pathways in cancer stem cells: clinical update. Nat Rev Clin Oncol. 2015;12:445–64. 25850553 10.1038/nrclinonc.2015.61 PMC4520755

[68] Takebe N, Harris PJ, Warren RQ, Ivy SP. Targeting cancer stem cells by inhibiting Wnt, Notch, and Hedgehog pathways. Nat Rev Clin Oncol. 2011;8:97–106. 21151206 10.1038/nrclinonc.2010.196

[69] Sun T, Zhang J. ETV4 mediates the Wnt/β-catenin pathway through transcriptional activation of ANXA2 to promote hepatitis B virus-associated liver hepatocellular carcinoma progression. J Biochem. 2021;170:663–73. 34347084 10.1093/jb/mvab088

[70] Zeng S, Seifert AM, Zhang JQ, Cavnar MJ, Kim TS, Balachandran VP, et al. Wnt/β-catenin signaling contributes to tumor malignancy and is targetable in gastrointestinal stromal tumor. Mol cancer therapeutics. 2017;16:1954–66. 10.1158/1535-7163.MCT-17-0139 PMC5587376 28611108

[71] Zheng H, Li W, Wang Y, Liu Z, Cai Y, Xie T, et al. Glycogen synthase kinase-3 beta regulates Snail and β-catenin expression during Fas-induced epithelial-mesenchymal transition in gastrointestinal cancer. Eur J Cancer. 2013;49:2734–46. 23582741 10.1016/j.ejca.2013.03.014

[72] Gan XQ, Wang JY, Xi Y, Wu ZL, Li YP, Li L. Nuclear Dvl, c-Jun, beta-catenin, and TCF form a complex leading to stabilization of beta-catenin-TCF interaction. J cell Biol. 2008;180:1087–100. 18347071 10.1083/jcb.200710050 PMC2290839

