# Supplementary material for: The pathogenic germline ETV4 P433L mutation identified in multiple primary lung cancer affect tumor stem-like property by Wnt/β-catenin pathway
Source: Cell Death Dis. 2024 Oct 10;15(10):738. doi: 10.1038/s41419-024-07129-z (PMC11467305; doi:10.1038/s41419-024-07129-z)
Supplement: Supplementary file 1 — Supplementary Material [file 41419_2024_7129_MOESM1_ESM.pdf]

Figure 3A-3B
A549-ETV4

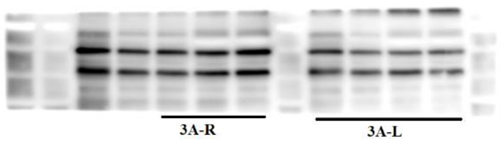

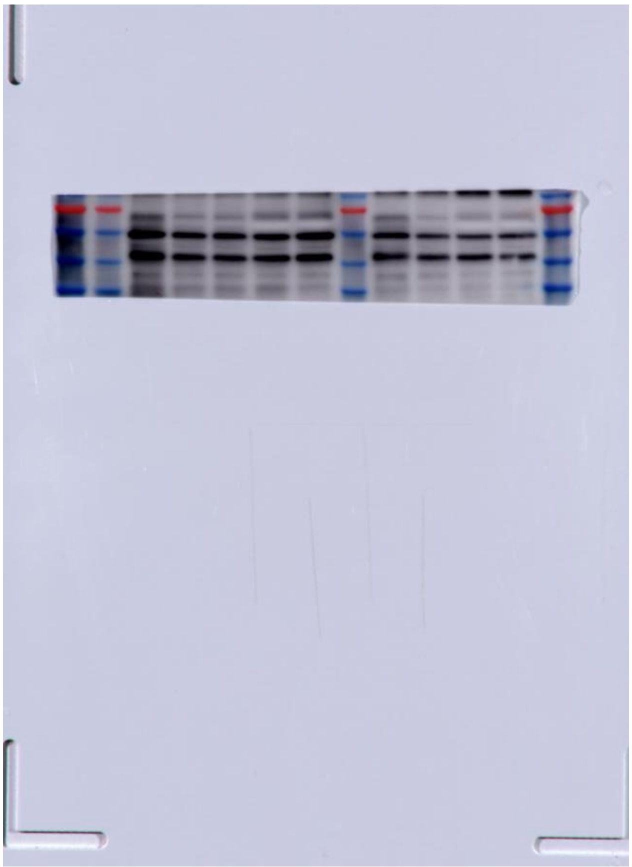

A549-β-actin

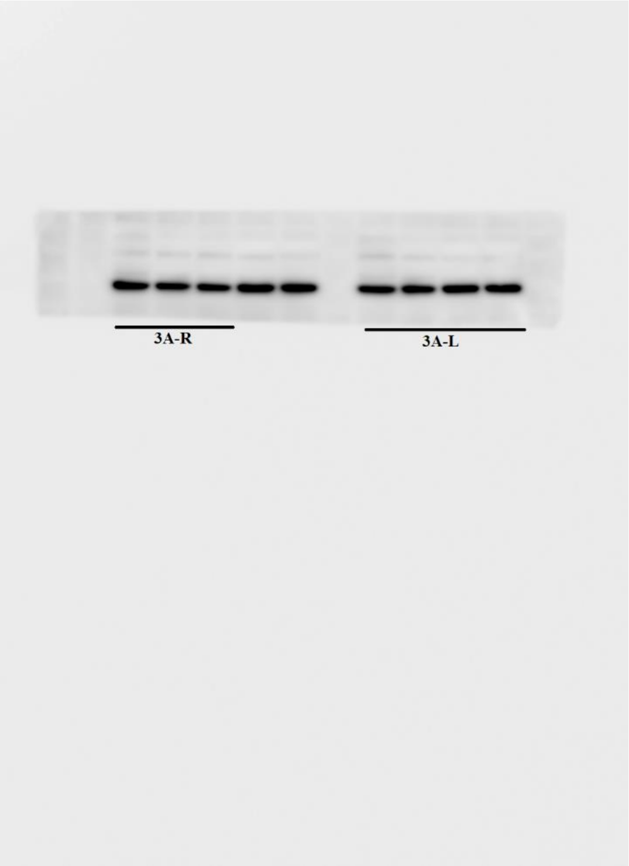

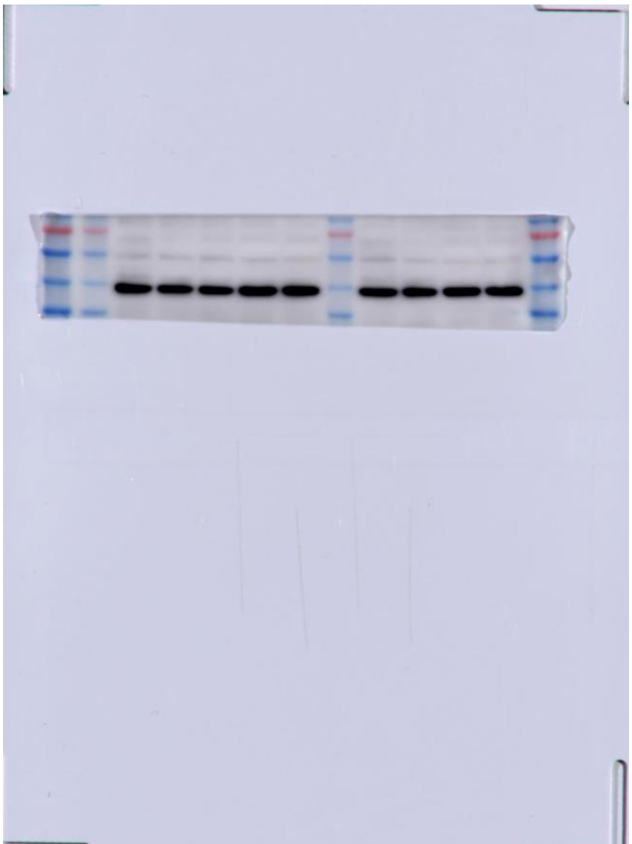

Figure 3B
H322-ETV4

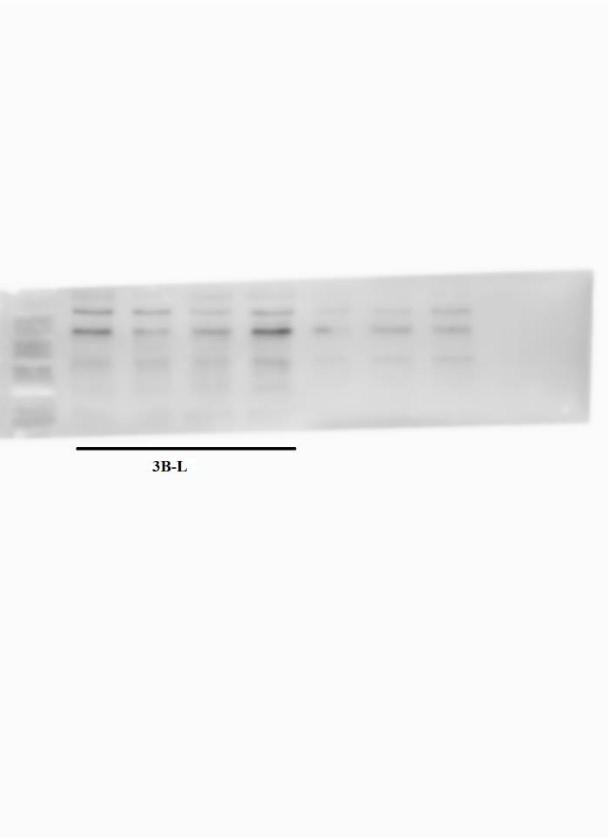

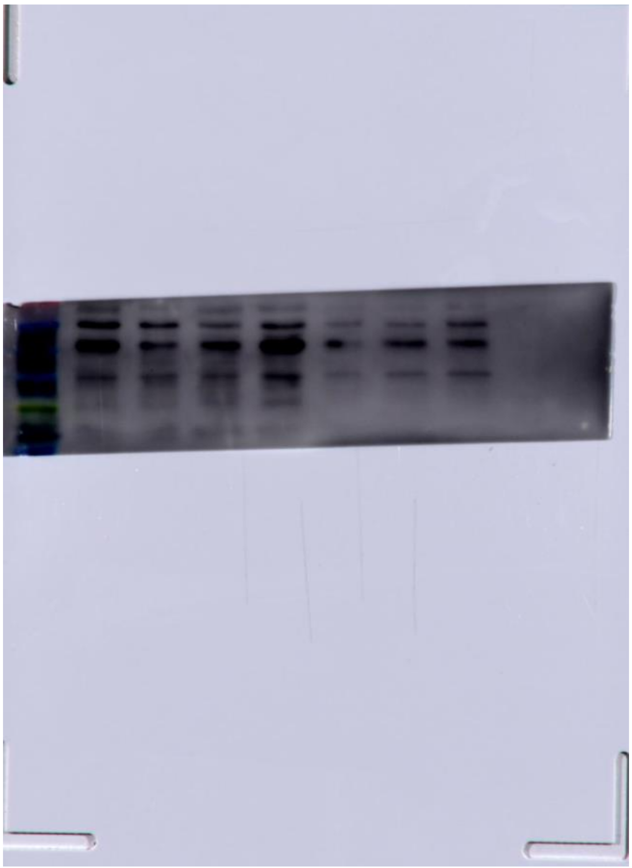

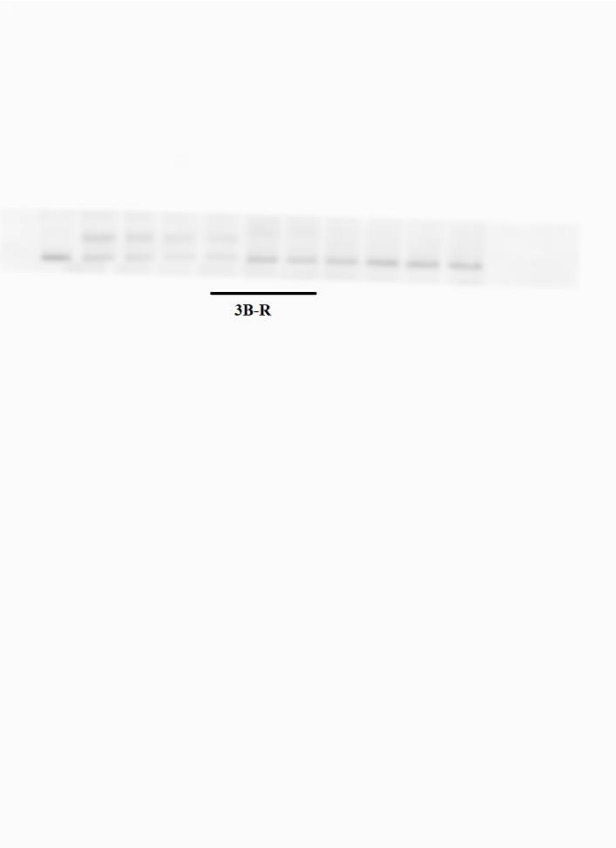
3B-R

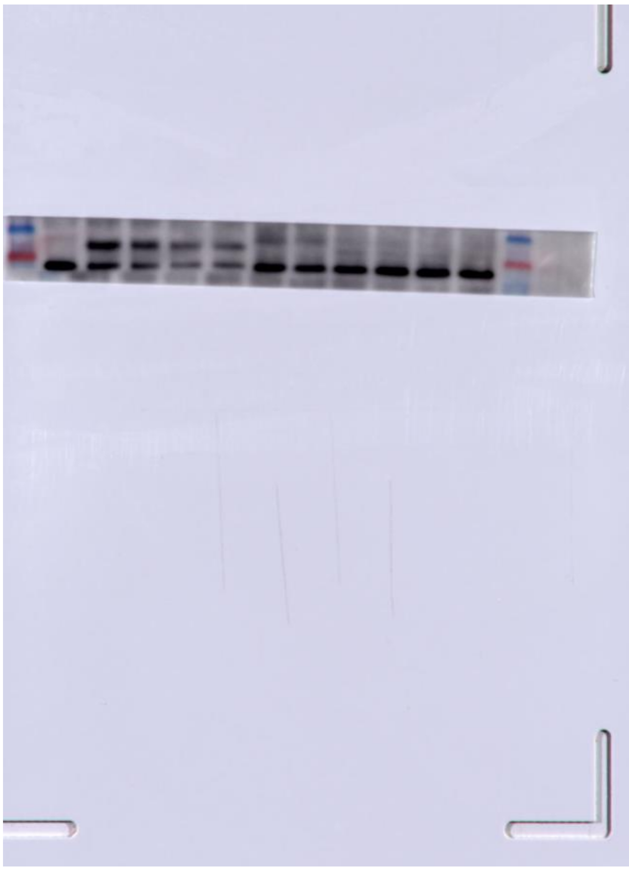

H322-β-actin

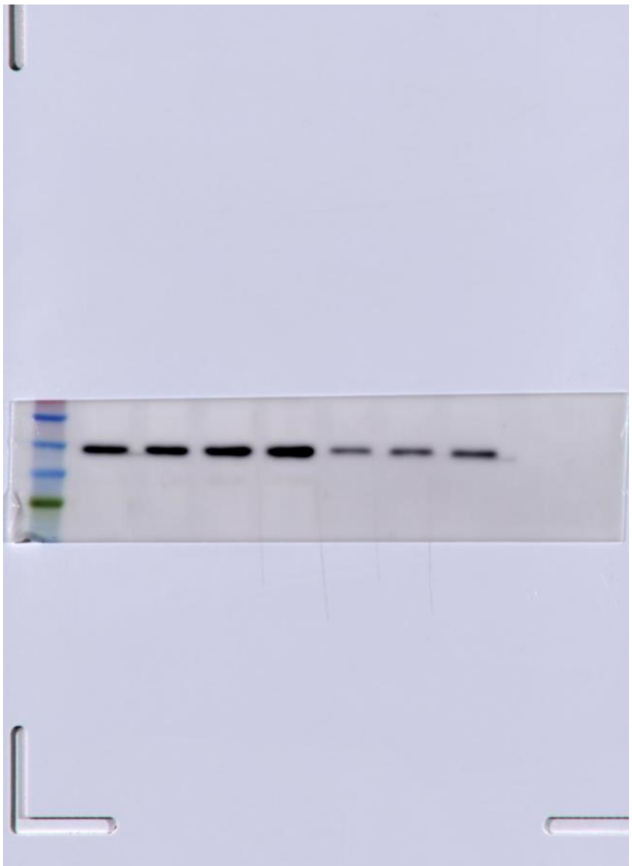

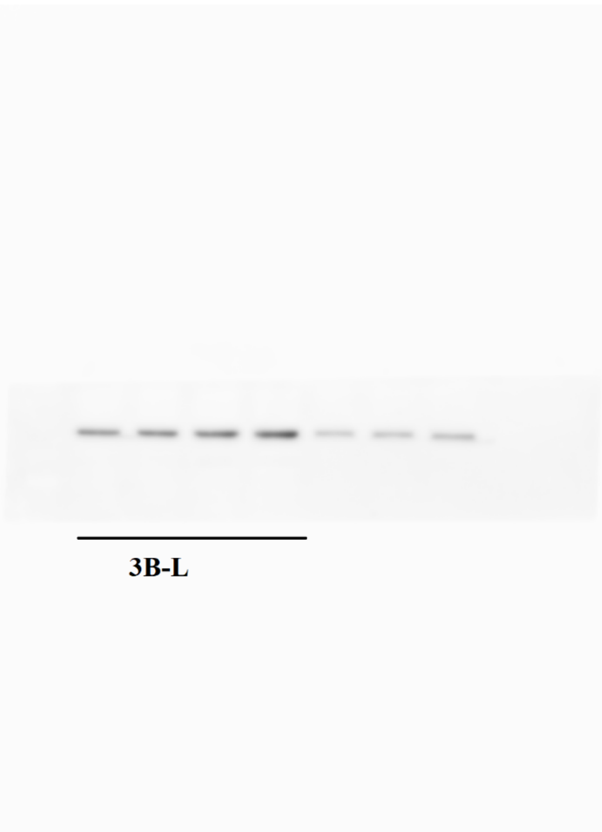

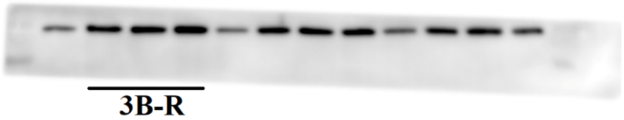

3B-R

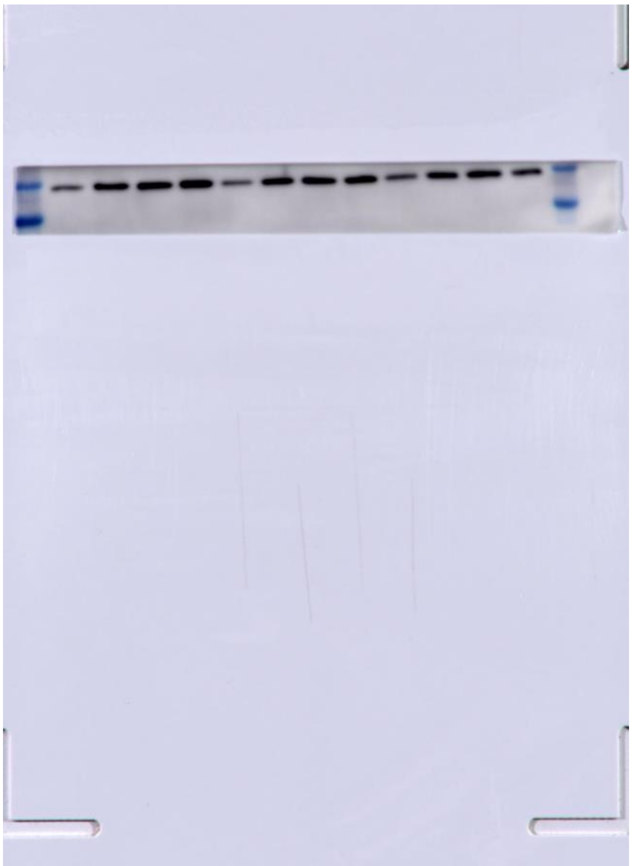

Figure 4E
ABCG2

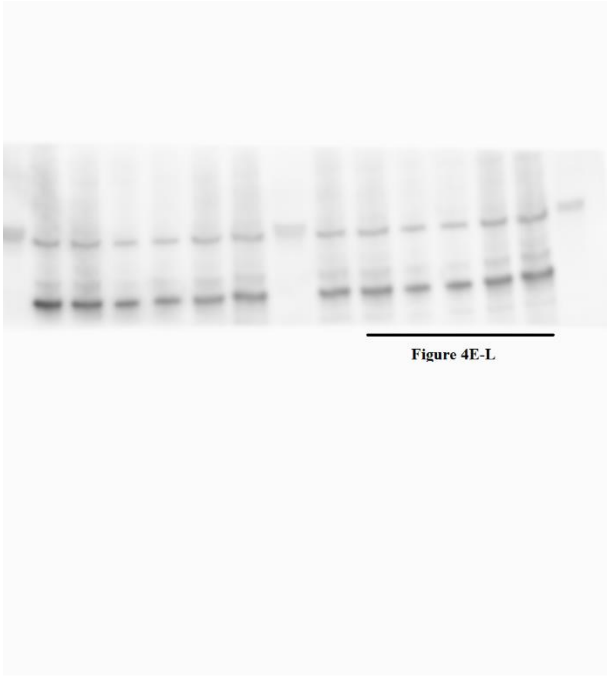

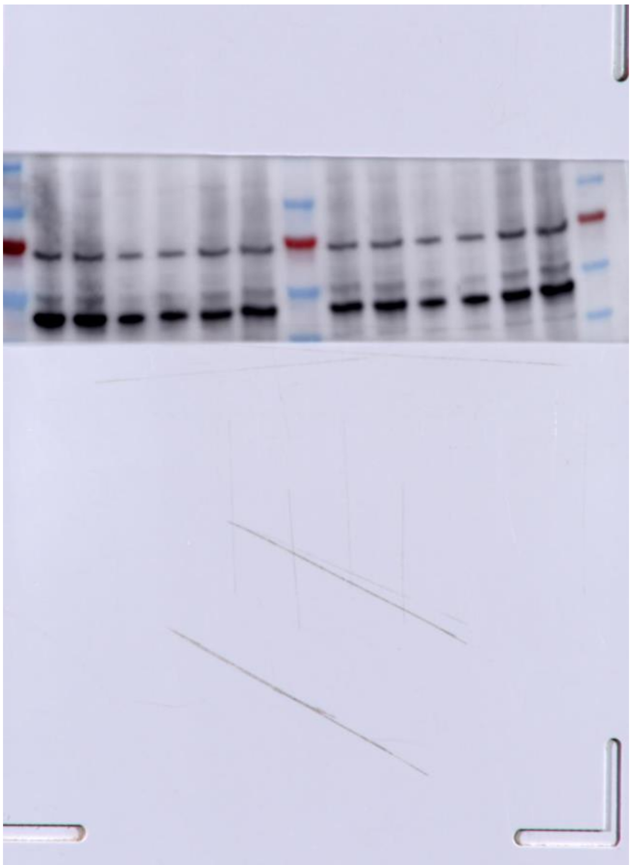

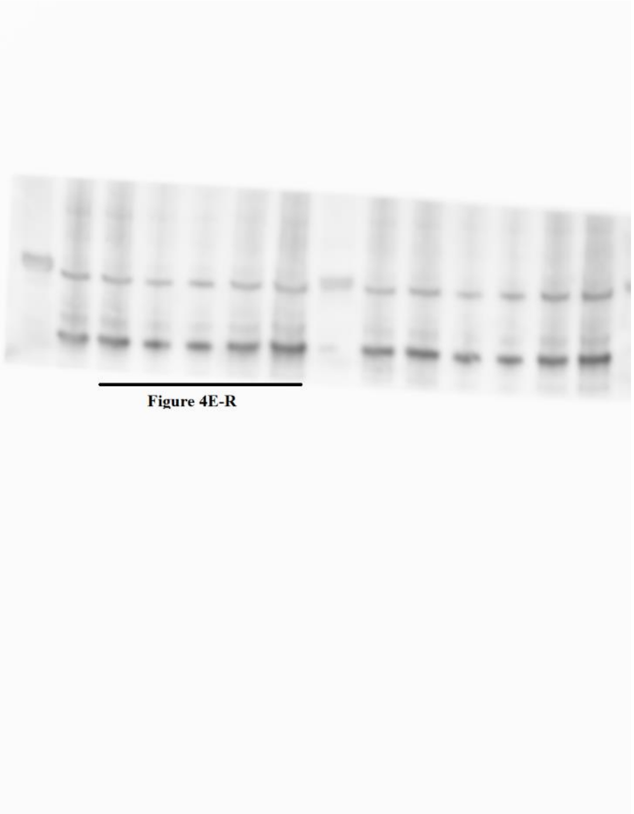

Figure 4E-R

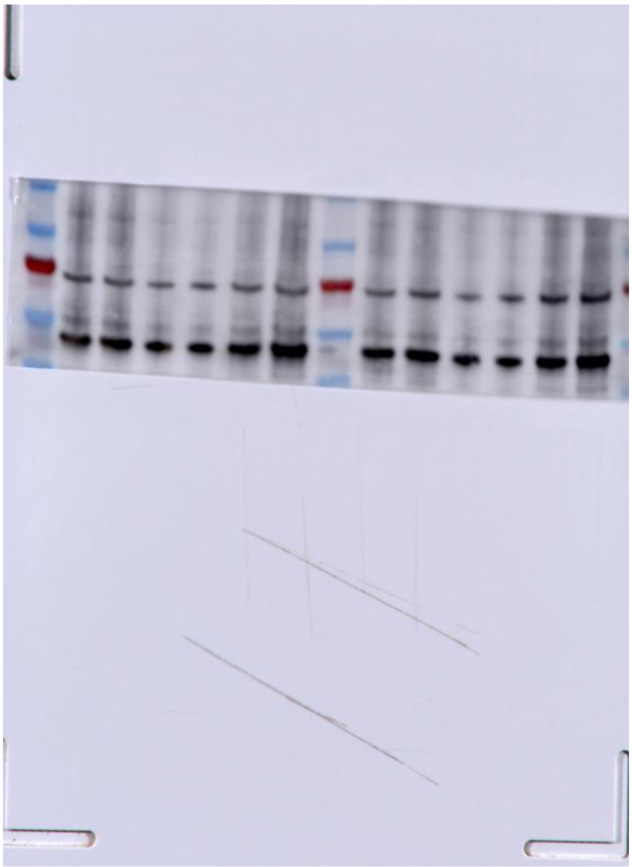

KLF4

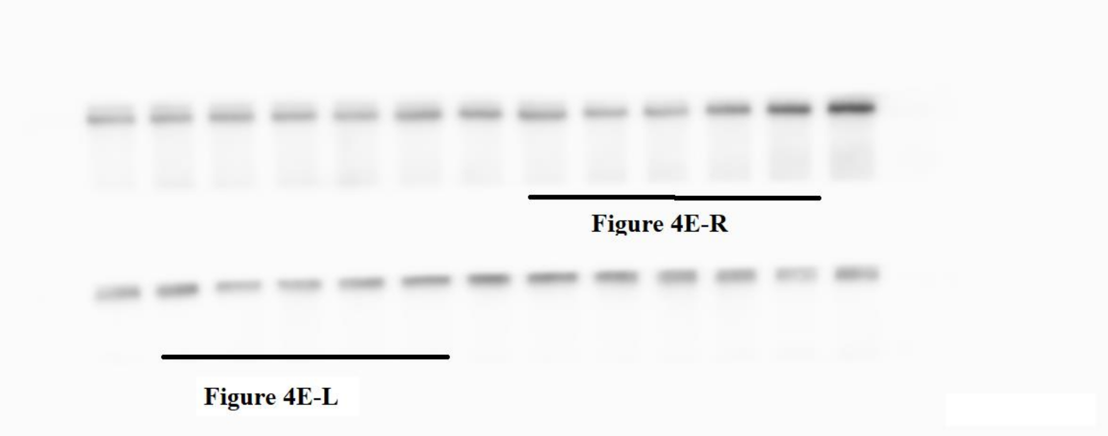

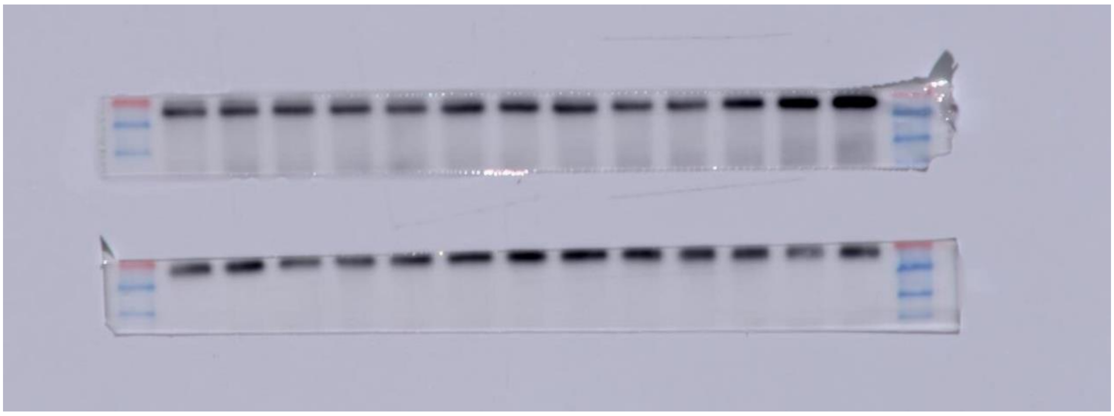

β-actin

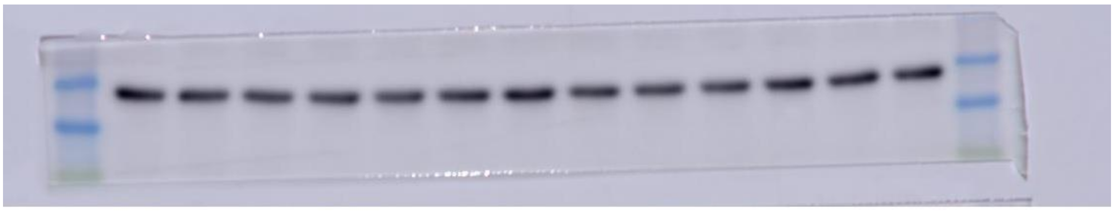

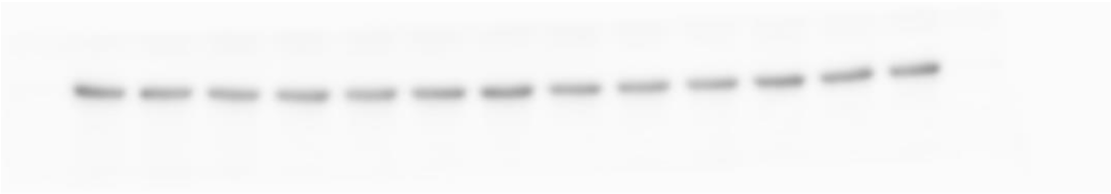

Figure 4E-L

Figure 4E-R

Figure 5E
ABCG2

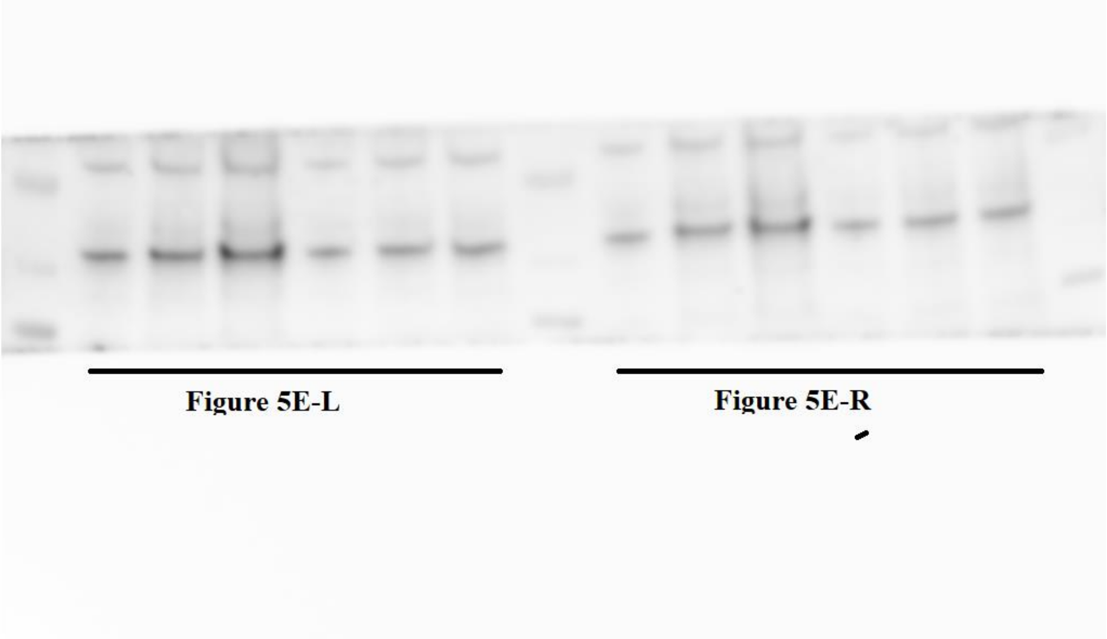

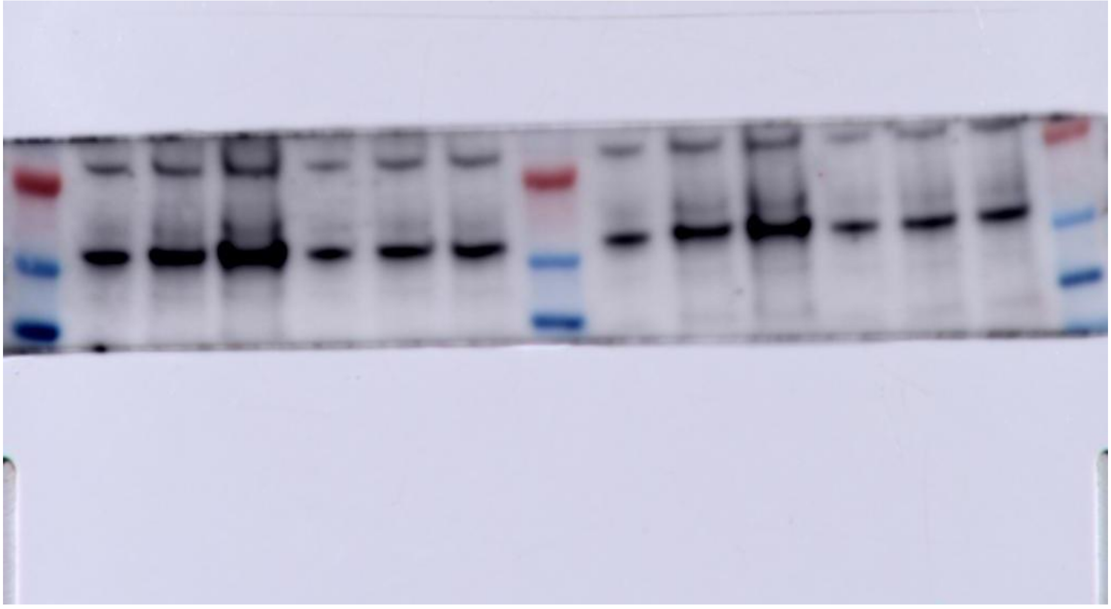

KLF4

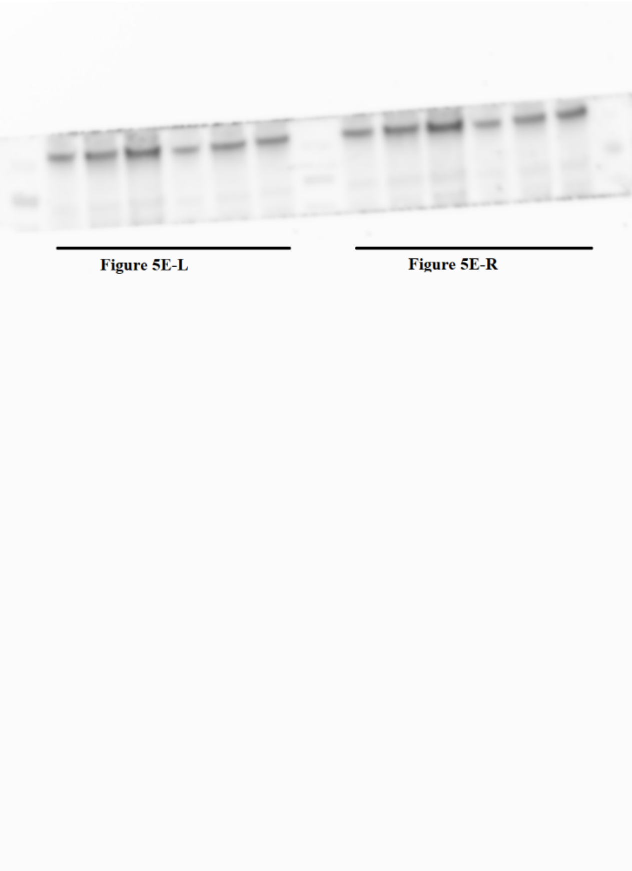

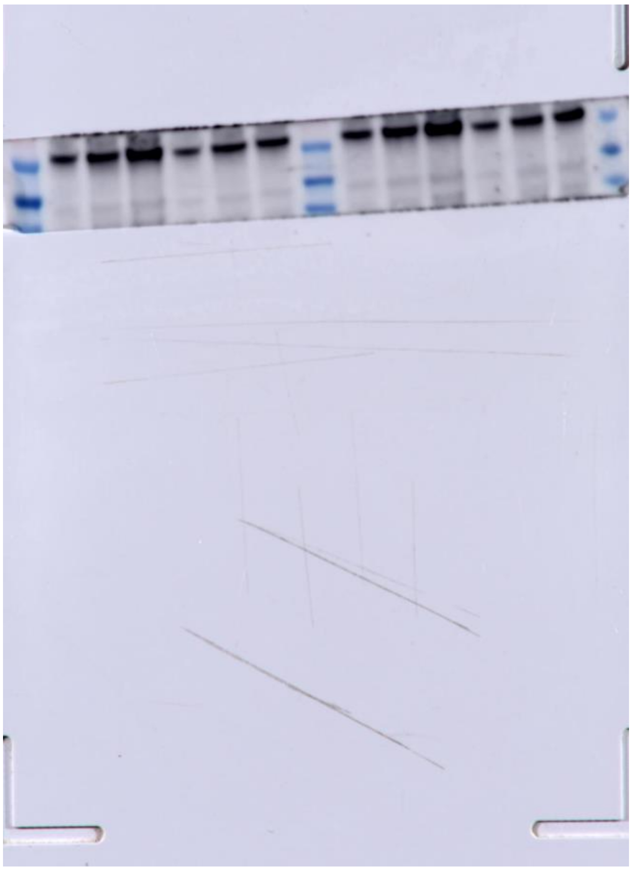

c-MYC

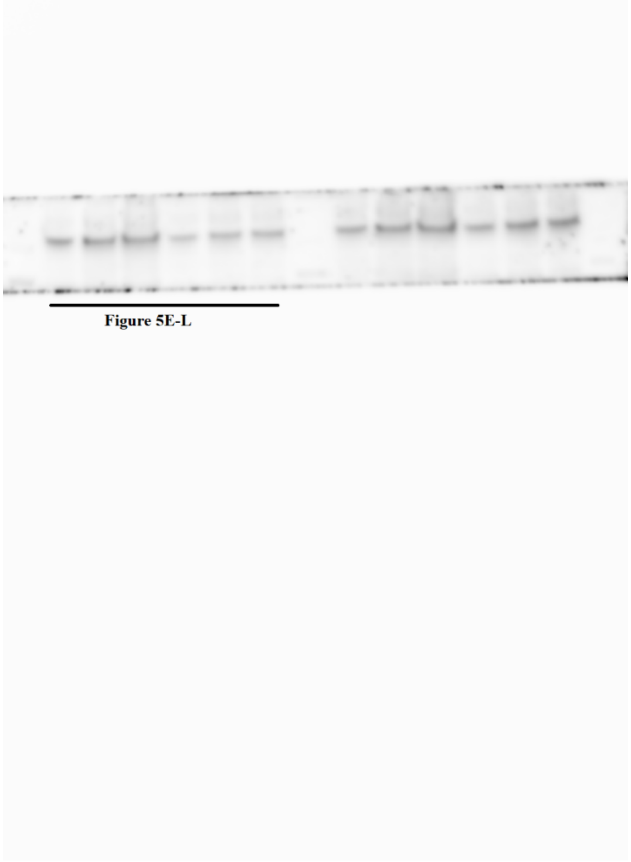

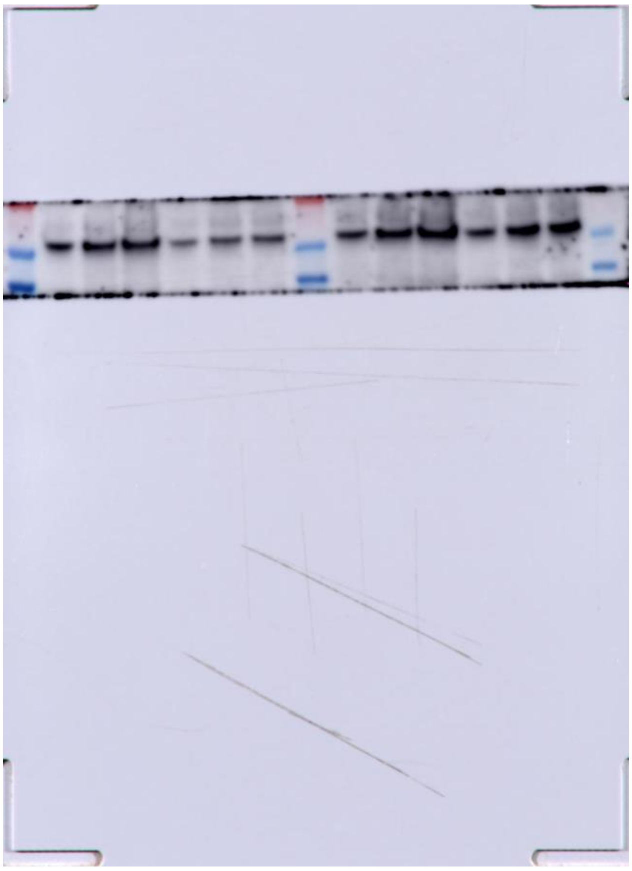

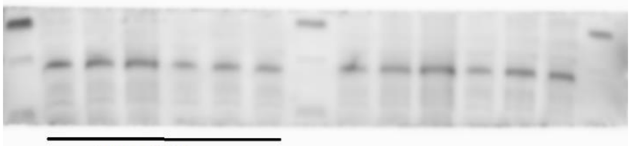

Figure 5E-R

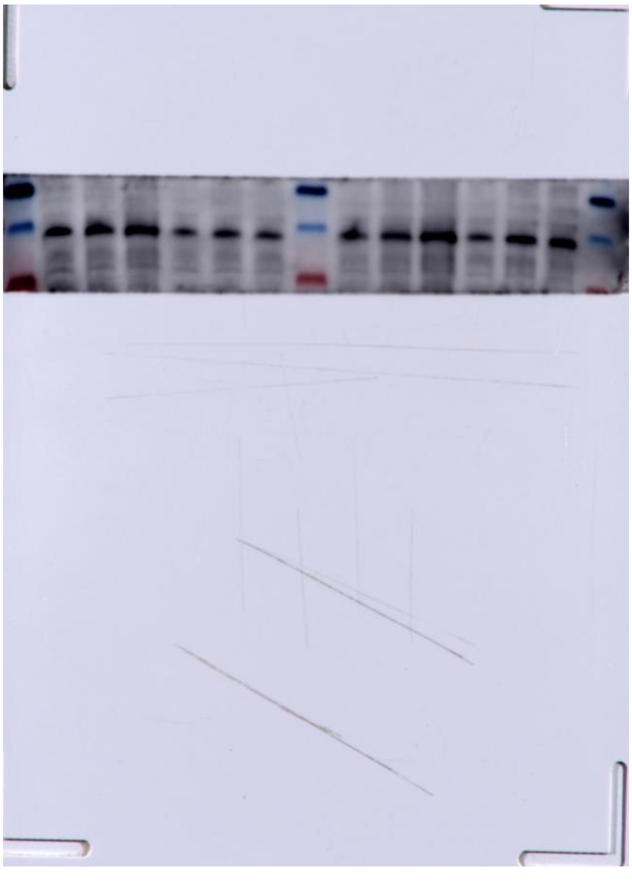

β-catenin

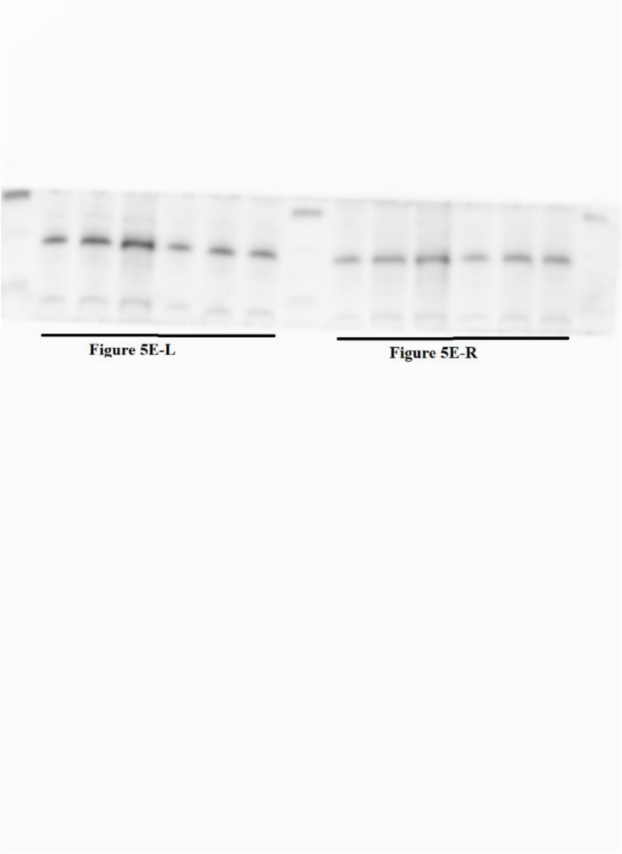

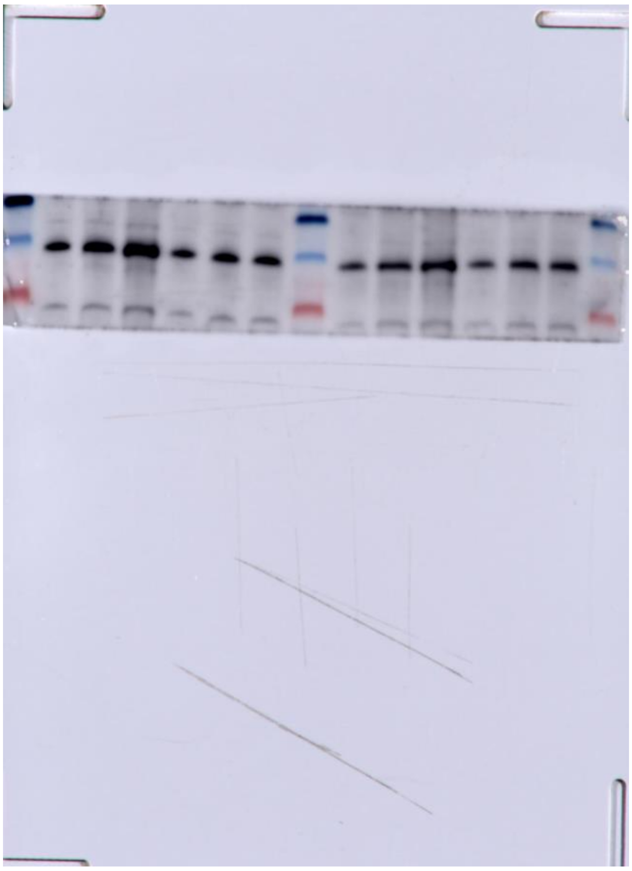

β-actin

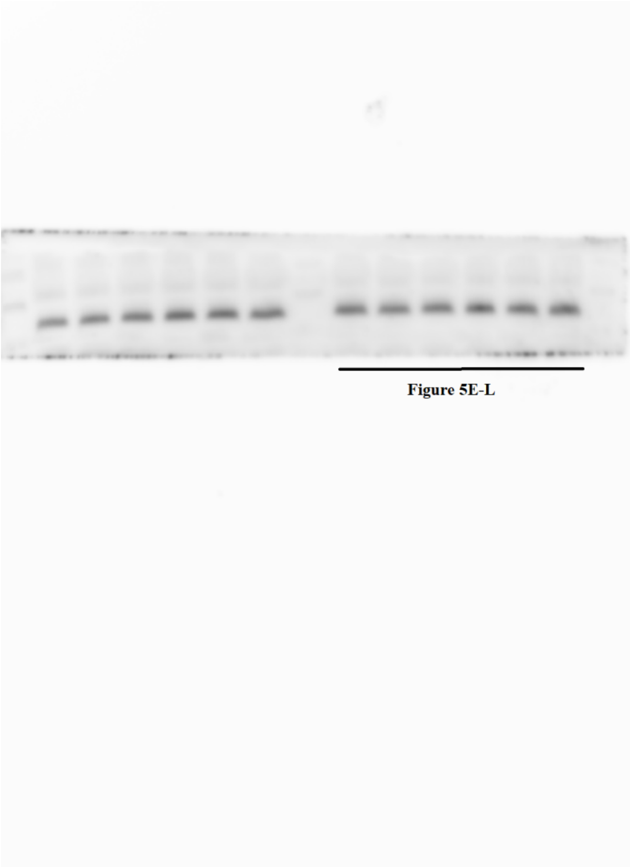

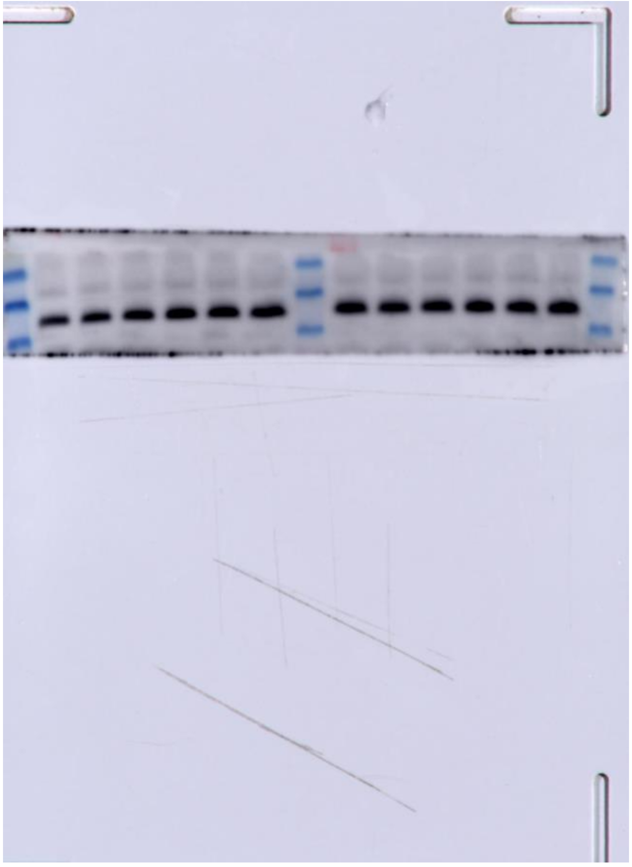

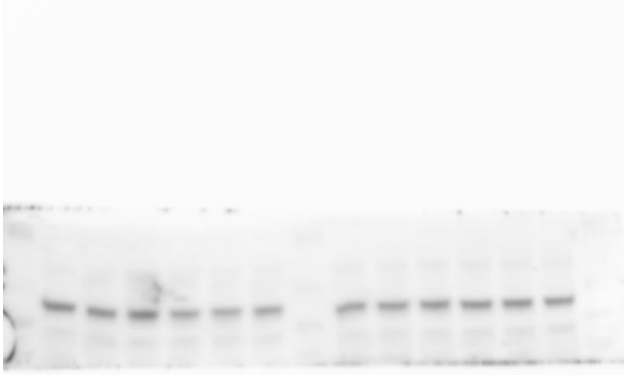

Figure 5E-R

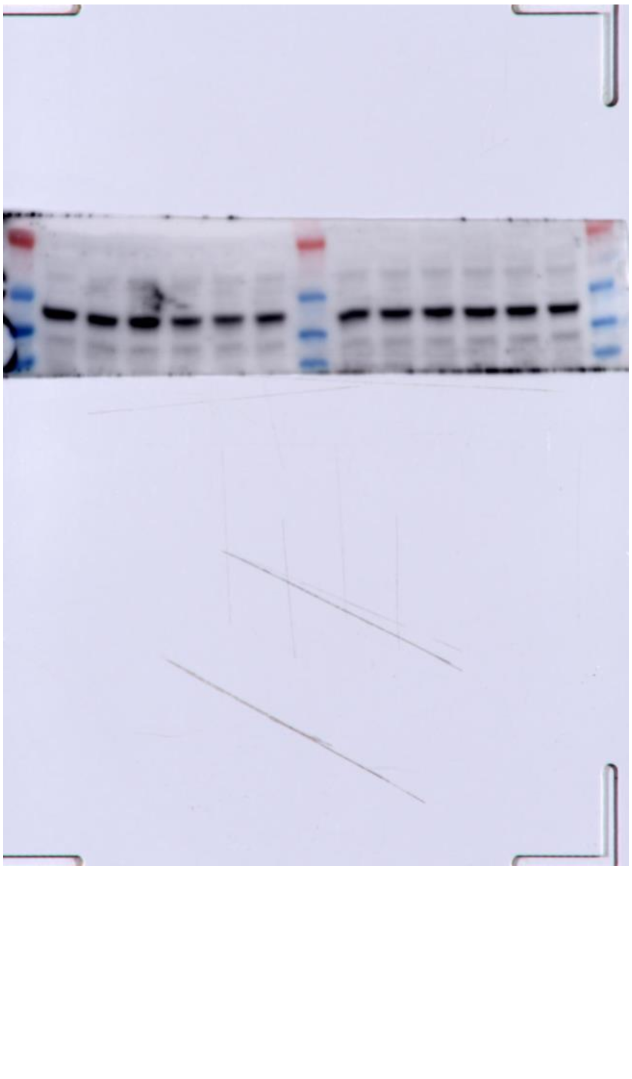

Stemness related gene list

ABCB11
ABCG2
ACHE
ADIPOR2
ADORA2A
ADRA1A
ADRA1B
ADRA1D
ADRA2A
ADRA2B
ADRA2C
ADRB1
ADRB2
ADRB3
AKT1
APOA1
AQP9
AR
ATP1A1
BACE1
BCHE
BCL2
BCL2L1
BCL2L2
CACNA1A
CACNA1C
CACNA1H
CACNA1I
CACNA2D2
CACNG1
CALM1
CALY
CAMK2A
CAMLG
CHEK1
CHRM1
CHRM2
CHRM3
CHRM4
CHRM5
CHRNA7
CLCN3

CPT1A
CPT2
CSNK2A1
CTGF
CYCS
CYP19A1
CYP1B1
CYP2B6
CYP2C19
CYP2C8
CYP3A4
CYP3A5
CYP3A7
CYP51A1
DHFR
DHFRP1
DNMT1
DRD1
DRD2
DRD3
DRD4
DRD5
EGF
ESR2
FOXM1
FPR1
GLO1
GPR35
GRIN1
GRIN2A
GRIN2B
GRIN2C
GRIN2D
GRIN3A
GSK3B
GSTP1
HBA1
HDAC1
HDAC10
HDAC11
HDAC2
HDAC3
HDAC4
HDAC5

HDAC6
HDAC7
HDAC8
HDAC9
HEXA
HRH1
HSD17B1
HSP90AA1
HTR1A
HTR1D
HTR1E
HTR2A
HTR2B
HTR2C
HTR6
HTR7
IKBKB
JUN
KCNA10
KCNA7
KCNH2
KCNJ10
KCNN4
KCNQ1
KCNQ4
LCK
LIPE
MALT1
MAOA
MAPK1
MAPK11
MAPK12
MAPK14
MAPK8
MCL1
MME
MTOR
NHP2L1
NPR2
NPY1R
NPY2R
NQO2
NR1I2
NR1I3

OPRD1
OPRK1
OPRM1
PANX1
PDE1A
PDE1B
PDE2A
PDE3A
PDE4A
PDE4B
PDE4C
PDE4D
PDE5A
PDE9A
PGR
PGRMC1
PIK3CA
PIK3CB
PIK3CD
PIK3CG
PLK1
POMC
PPIA
PPID
PPIF
PPP3CA
PPP3R2
PRKCA
PRKCD
PRKDC
PTGS1
PTGS2
RELA
ROCK1
RPL10L
RPL11
RPL13A
RPL15
RPL19
RPL23
RPL23A
RPL26L1
RPL3
RPL37

RPL8
RPS6KB1
RSL24D1
S100A4
SCN4A
SCN5A
SCN9A
SGK1
SIGMAR1
SIRT1
SLC10A1
SLC12A1
SLC12A2
SLC23A1
SLC47A1
SLC6A2
SLC6A3
SLC6A4
SLCO1B1
SLCO1B3
SMPD1
STAT3
TGM2
TNNC1
TOP1
TOP1MT
TRPM2
TRPM4
TRPM8
TRPV5
TUBA1A
TUBB
TUBD1
TUBE1
TUBG1
TXNRD1
TXNRD2
XDH
SOX2
NANOG
KLF4
CD133
ALDH1
BMI1

CD44
OCT4
