# Supplementary material for: The pathogenic germline ETV4 P433L mutation identified in multiple primary lung cancer affect tumor stem-like property by Wnt/β-catenin pathway
Source: Cell Death Dis. 2024 Oct 10;15(10):738. doi: 10.1038/s41419-024-07129-z (PMC11467305; doi:10.1038/s41419-024-07129-z)
Supplement: Supplementary file 2 — Supplementary Figure [file 41419_2024_7129_MOESM2_ESM.pdf]

A

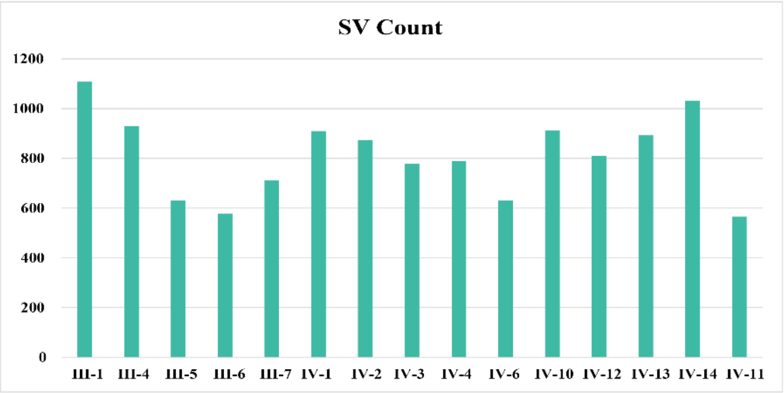

B

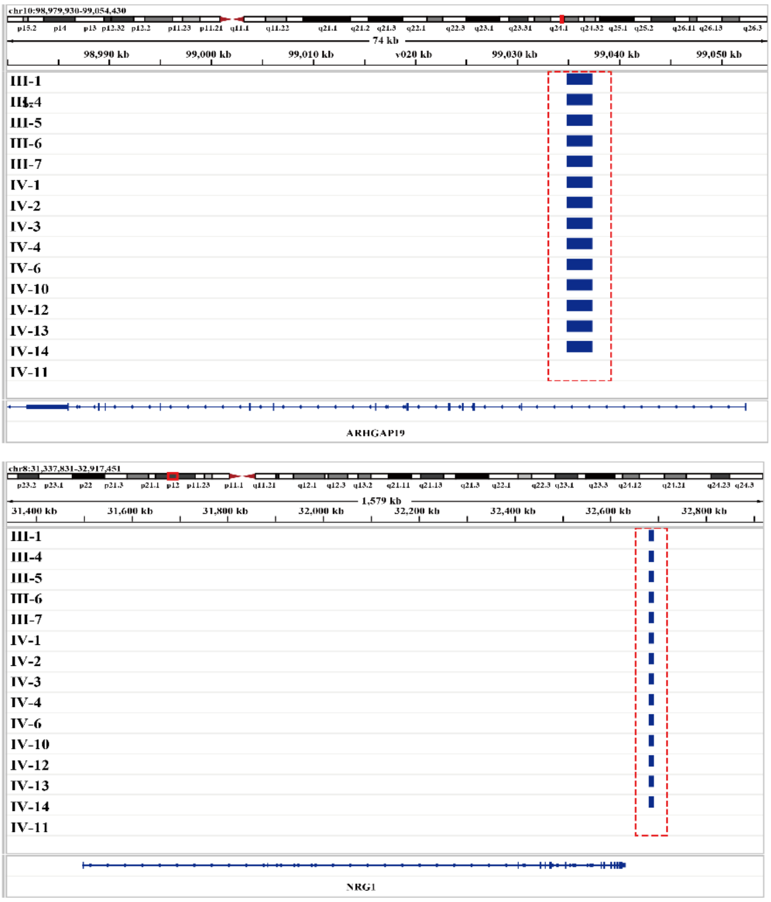

Supplementary Fig 1. The whole genome sequencing (WGS) results for these variations. (A) The SV count of the variation was showed in each sample. **(B)** A total of 2 mutation events (ARHGAP19 and NRG1) were identified by applying screening criteria of rec = 5 in the lung cancer group and rec = 9 in the nodule group.

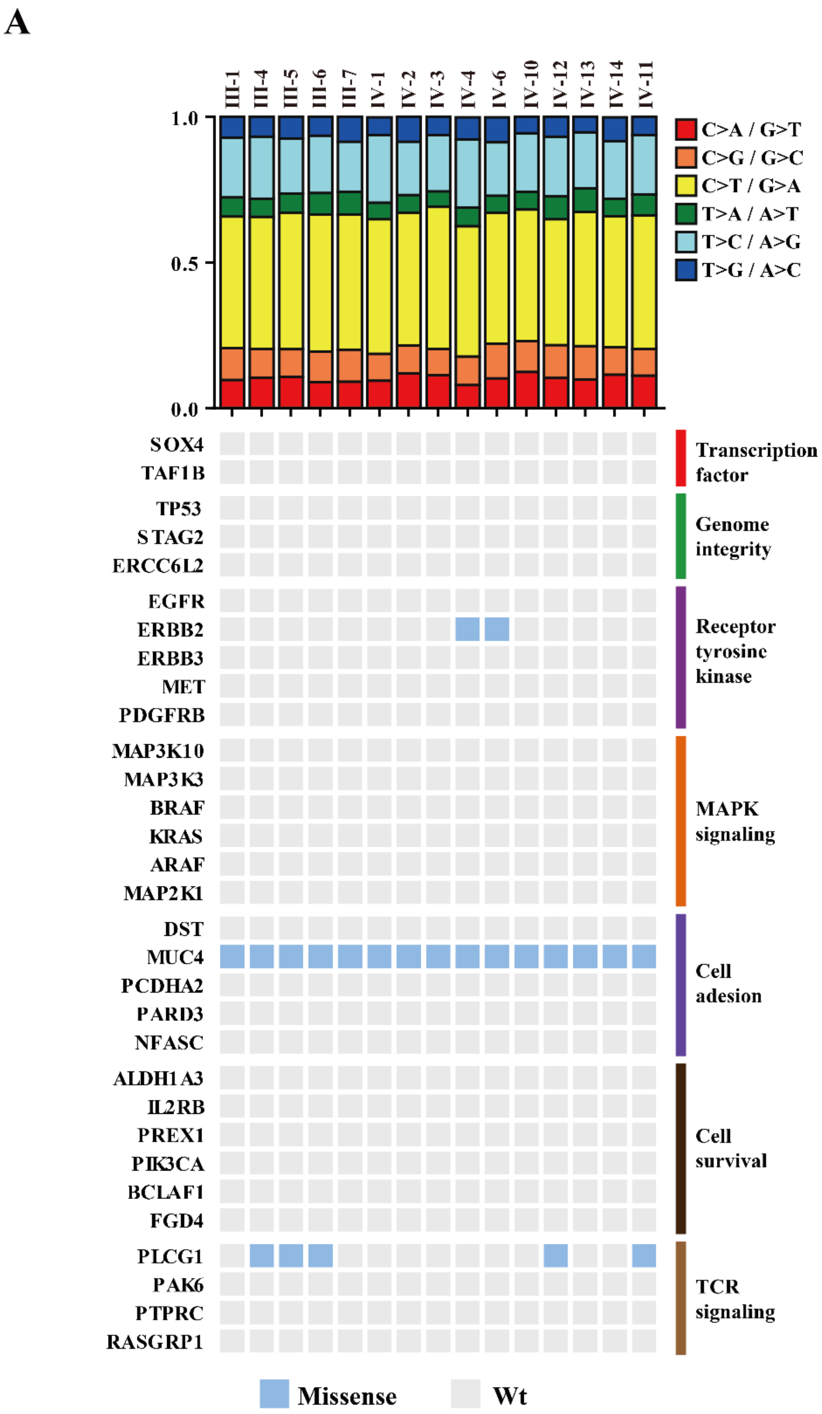

Supplementary Fig 2. The enrichment of common driver gene pathways and results was showed.

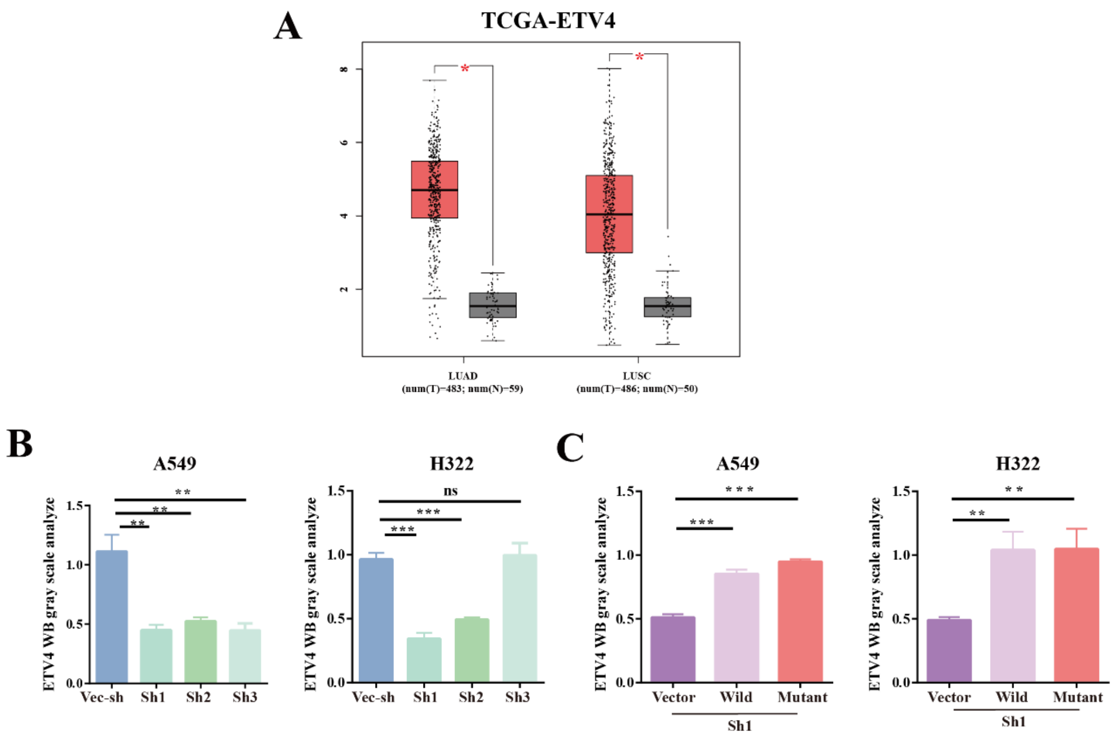

Supplementary Fig 3. The expression of ETV4 in TCGA and ETV4 protein expression gray scale analysis in A549 and H322. (**A**) The ETV4 expression is showed in LUAD and LUSC from TCGA. (**B-C**) The ETV4 WB expression gray scale analysis results in A549 and H322 knockdown and overexpression cell lines. NS means no significance.

*p<0.05, **p<0.01, ***p<0.001

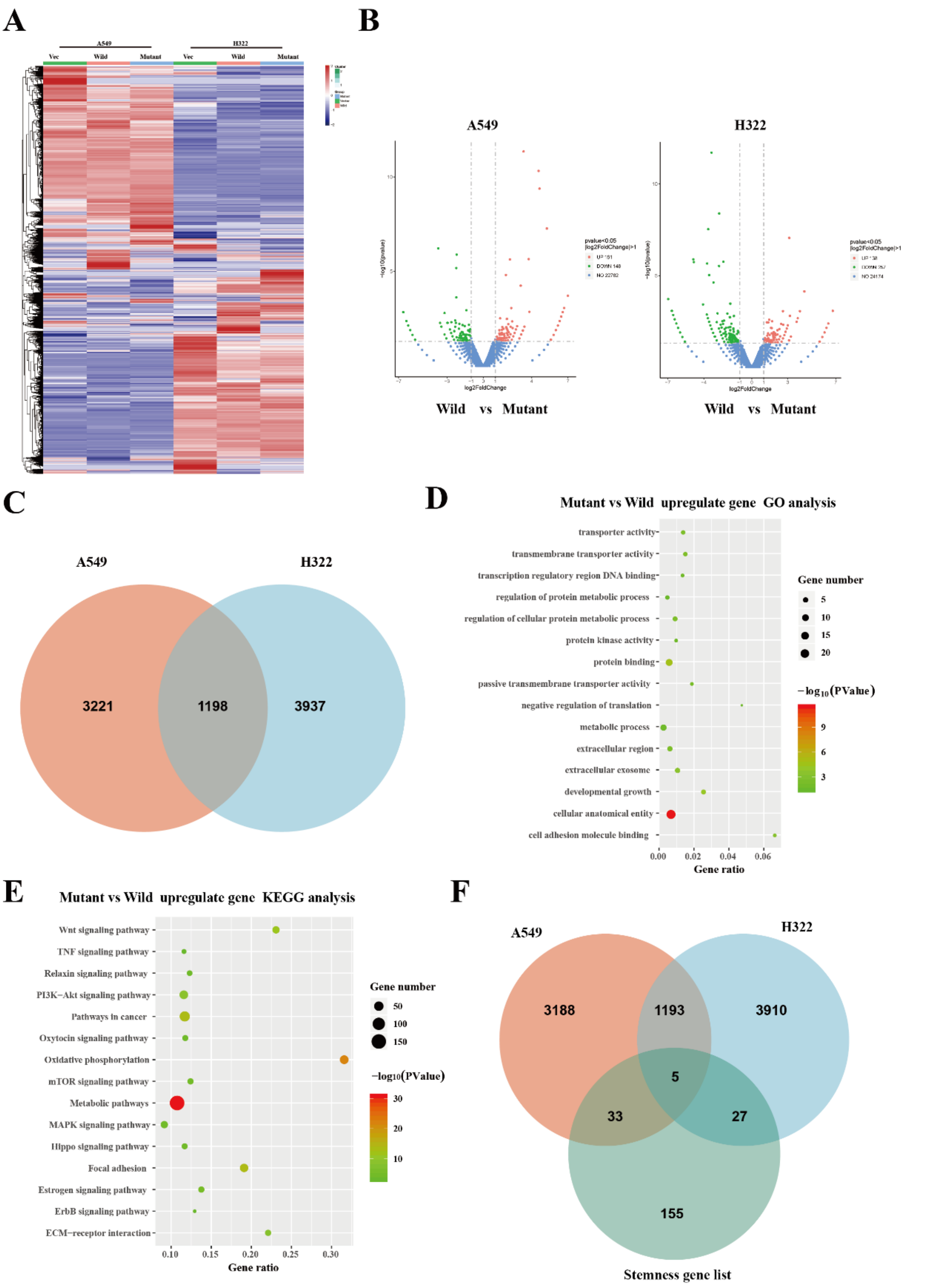

Supplementary Fig 4. The differential genes and function enrichment analysis results through RNA-seq analysis in A549 and H322 cell lines. **(A)** The heatmap shows the gene expression of *ETV4* overexpression (vector, wild and mutant) group in shETV4-1 A549

and H322 cell lines by RNA-seq. **(B)**The volcano shows the differential genes between wild and mutant cell lines in A549 and H322. **(C)**The Venn diagram shows the intersection of different gene between wild and mutant cell lines in A549 and H322. **(D)**The GO functional enrichment analysis of the high expression gene between mutant and wild group differential gene in A549 and H322. **(E)**The KEGG pathway enrichment analysis of the high expression gene between mutant and wild group differential gene in A549 and H322. **(F)**The Venn diagram shows the intersection of wild and mutant group differential genes in A549 and H322 cell lines, and the intersection of stemness related gene.

*$p<0.05$, **$p<0.01$, ***$p<0.001$

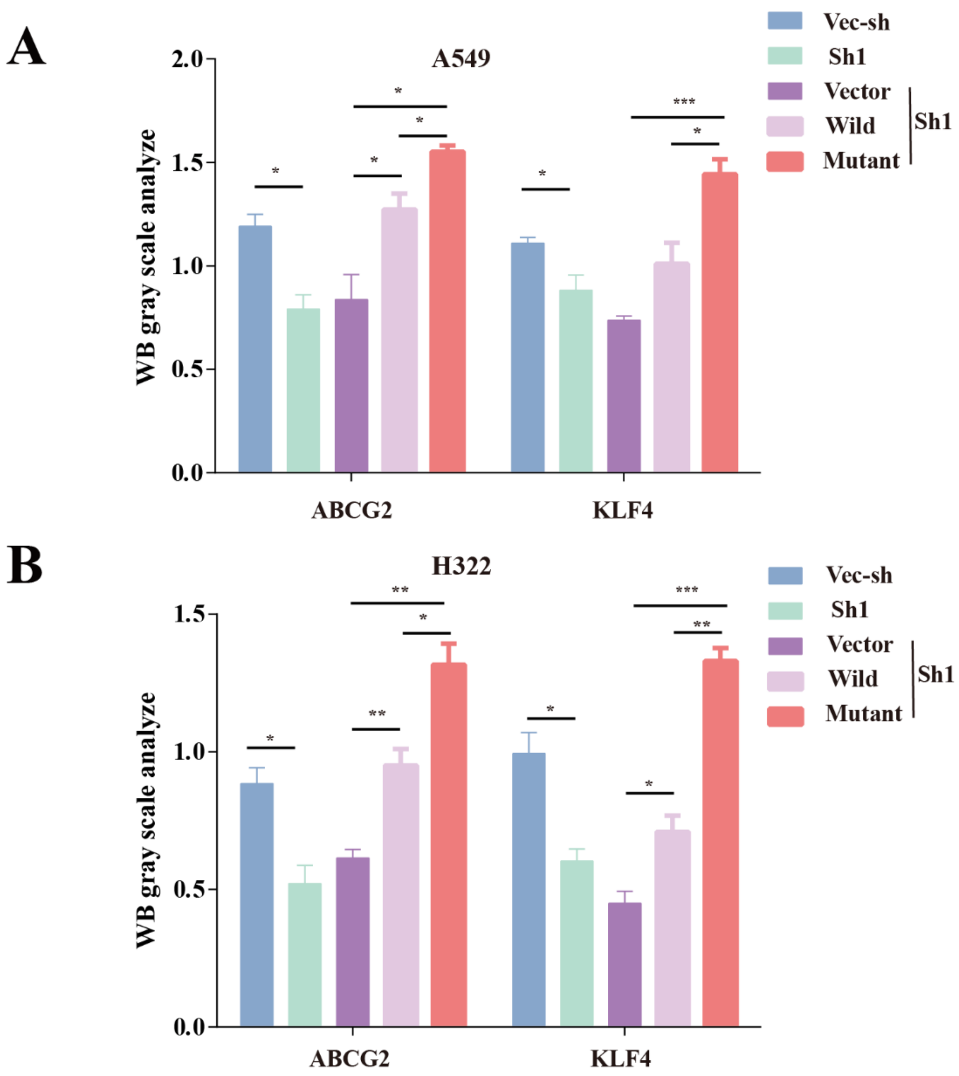

Supplementary Fig 5. The ABCG2 and KLF4 protein expression gray scale analysis in A549 and H322. **(A)** The ABCG2 and KLF4 protein expression gray scale analysis results in A549 knockdown (vec-sh and sh1) and overexpression (vector, wild and mutant) cell lines. **(B)** The ABCG2 and KLF4 protein expression gray scale analysis results in H322 knockdown (vec-sh and sh1) and overexpression (vector, wild and mutant) cell lines.*p<0.05, **p<0.01, ***p<0.001

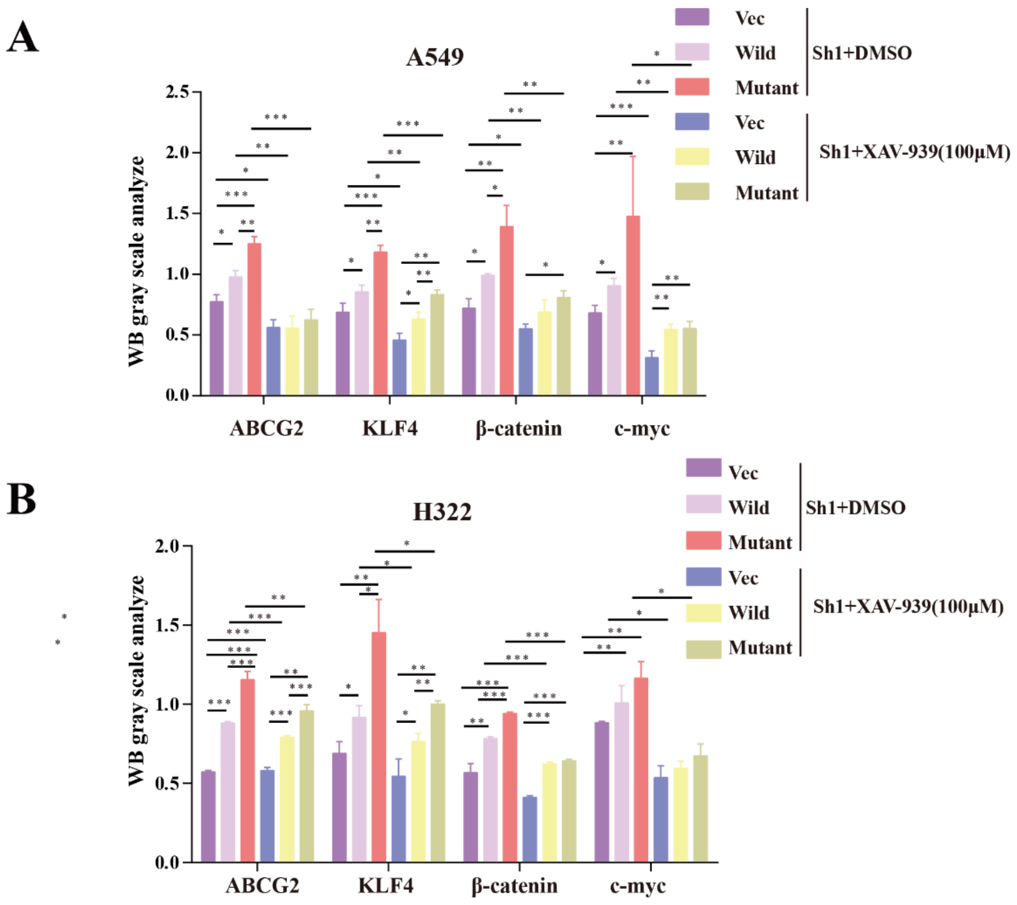

Supplementary Fig 6. The stemness marker and Wnt/β-catenin signaling protein expression gray scale analysis in A549 and H322. (A-B) The gray scale analysis of stemness markers ABCG2, KLF4 and β-catenin and c-Myc in vector, wild-type and ETV4 P433L mutation cells (vector, wild and mutant) were measured in the presence or absence of 100 μM XAV-939 in A549 and H322 cell lines. Three independent biological replicates were conducted for *in vitro* assays. Data are presented as mean ± SD. *p<0.05, **p<0.01, ***p<0.001

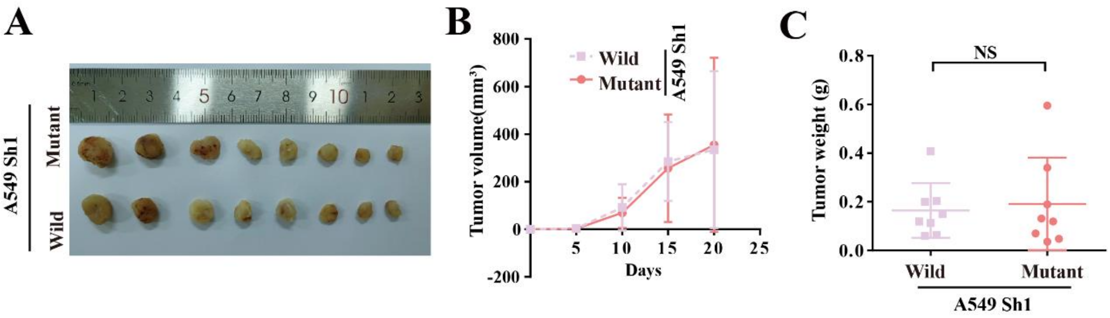

Supplementary Fig 7. In vitro experiments showed no significant difference in xenogenic subcutaneous tumor formation between the mutant and wild groups. (A) Images of xenograft tumors with ETV4 overexpression (wild and mutant) in shETV4-1 A549 cell lines. **(B-C)** Volumes and weights of these xenograft tumors. NS means no significance.
