# Supplementary material for: The pathogenic germline ETV4 P433L mutation identified in multiple primary lung cancer affect tumor stem-like property by Wnt/β-catenin pathway
Source: Cell Death Dis. 2024 Oct 10;15(10):738. doi: 10.1038/s41419-024-07129-z (PMC11467305; doi:10.1038/s41419-024-07129-z)
Supplement: Supplementary file 3 — Supplementary Table [file 41419_2024_7129_MOESM3_ESM.pdf]

Supplementary Table 1. The gene primers sequence.

Gene	Primers sequence
β-Actin-F	CATGTACGTTGCTATCCAGGC
β-Actin-R	CTCCTTAATGTCACGCACGAT
ETV4-F	ACCCTCCTGCCAGTATGAA
ETV4-R	CCGAGCATCTGCCTGTAC
SOX2-F	GCCGAGTGGAAACTTTTGTCG
SOX2-R	GGCAGCGTGTACTTATCCTTCT
NANOG-F	TTTGTGGGCCTGAAGAAAACT
NANOG-R	AGGGCTGTCCTGAATAAGCAG
KLF4-F	CCCACATGAAGCGACTTCCC
KLF4 -R	CAGGTCCAGGAGATCGTTGAA
CD133-F	AGTCGGAAACTGGCAGATAGC
CD133-R	GGTAGTGTTGTACTGGGCCAAT
ALDH1-F	TTGGAATTTCCCGTTGGTTA
ALDH1-R	CTGTAGGCCCATAACCAGGA
BMI1-F	CCACCTGATGTGTGTGCTTTG
BMI1-R	TTCAGTAGTGGTCTGGTCTTGT
ABCG2-F	CAGGTGGAGGCAAATCTTCGT
ABCG2-R	ACCCTGTTAATCCGTTCGTTTT
CD44-F	CTGCCGCTTTGCAGGTGTA
CD44-R	CATTGTGGGCAAGGTGCTATT
OCT4-F	CTGGGTTGATCCTCGGACCT
OCT4-R	CCATCGGAGTTGCTCTCCA
β-catenin-F	AAAGCGGCTGTTAGTCACTGG
β-catenin -R	CGAGTCATTGCATACTGTCCAT
c-Myc-F	GGCTCCTGGCAAAAGGTCA
c-Myc -R	CTGCGTAGTTGTGCTGATGT
